# Supplementary material for: Harmonizing across datasets to improve the transferability of drug combination prediction
Source: Commun Biol. 2023 Apr 11;6:397. doi: 10.1038/s42003-023-04783-5 (PMC10090076; doi:10.1038/s42003-023-04783-5)

Supplementary tables

Supplementary Table 1. Summary basic information of all datasets used in this study obtained from DrugComb. The numbers of total experiments, monotherapy drugs, drug pair combinations, cell lines, cell line-treatment combinations, and experimental settings (dose-response matrices and dose ranges) are shown in the table below.

Study	#experiment	# drug	#drug combination	#cell line	#cell line/-treatment combination	dose-response matrix	dose range (μm)
ALMANAC	311,604	103	5142	60	299,548	4×4 or 4×6	0~250
O'Neil	92,208	38	583	39	22,737	5×5	0~20
FORCINA	1,818	1818	1818	1	1,818	2×2	0~400
Mathews	1,119	477	967	1	967	6×6 or 10×10	0~1000

Supplementary Table 2. Performances (Pearson's r) of all models tested in this study in intra-study cross-validation. The best performances for each dataset, each score, were marked as red. The features for all models are listed in **Supplementary Table 5.**

	ALMANAC																			
S	0.7453	0.7396	0.7371	0.7222	0.772	0.7816	0.2574	0.4743	0.6048	0.771	0.7879	0.7949	0.6615	0.6608	0.8051	0.8353	0.8352	0.8357	0.8326	0.8413
ZIP	0.6527	0.6243	0.623	0.6016	0.6402	0.6627	0.1733	0.5051	0.5687	0.6413	0.6485	0.665	0.6833	0.6847	0.7522	0.7741	0.7751	0.776	0.7654	0.7752
Loewe	0.8369	0.6387	0.6377	0.7597	0.8095	0.8659	0.2718	0.4918	0.6943	0.8142	0.8463	0.8698	0.7085	0.709	0.8205	0.8651	0.8615	0.8615	0.8521	0.8753
HSA	0.5213	0.4519	0.4583	0.4154	0.4835	0.5326	0.1421	0.2727	0.4003	0.4905	0.5015	0.535	0.6872	0.6889	0.7075	0.7194	0.7186	0.7194	0.7046	0.7183
Bliss	0.5643	0.5071	0.5087	0.4728	0.5339	0.5737	0.1296	0.334	0.4459	0.5367	0.548	0.5758	0.6707	0.672	0.7256	0.7418	0.7415	0.7426	0.7297	0.7401
CSS	0.9205	0.9181	0.9177	0.9112	0.9268	0.9301	0.2712	0.8457	0.8756	0.9234	0.9285	0.9306	0.8813	0.8815	0.9314	0.9436	0.9435	0.9436	0.9422	0.9463
	O'Neil																			
S	0.9063	0.9018	0.8995	0.8892	0.9035	0.9087	0.5895	0.7756	0.8673	0.9047	0.9049	0.9091	0.9069	0.9071	0.9202	0.9235	0.9233	0.9233	0.9213	0.9218
ZIP	0.6608	0.6175	0.6165	0.5956	0.6307	0.6677	0.2478	0.3342	0.5654	0.6257	0.6347	0.6677	0.7297	0.7298	0.728	0.7343	0.7361	0.7322	0.7275	0.7318
Loewe	0.8618	0.7964	0.802	0.8271	0.8464	0.8727	0.4022	0.5958	0.7663	0.8489	0.863	0.8721	0.7988	0.7996	0.8489	0.8765	0.8752	0.8717	0.8748	0.8794
HSA	0.606	0.5655	0.5685	0.5442	0.577	0.6075	0.2478	0.3113	0.5224	0.5757	0.5851	0.6041	0.6916	0.6956	0.684	0.6942	0.6938	0.6849	0.688	0.6881
Bliss	0.6125	0.5745	0.5723	0.556	0.585	0.6134	0.2352	0.3191	0.5287	0.5864	0.5906	0.6159	0.703	0.7009	0.6922	0.6997	0.697	0.6985	0.698	0.7
CSS	0.9109	0.9055	0.9053	0.8987	0.9089	0.9128	0.5115	0.7838	0.8755	0.908	0.9099	0.9131	0.9193	0.9194	0.923	0.9263	0.9257	0.9257	0.9254	0.9258
	FORCINA																			
S	0.6301	0.6272	0.6272	0.6221	0.6274	0.6274	−0.0428	0.6297	0.6289	0.6267	0.6268	0.6268	−0.0432	0.2074	0.7218	0.7218	0.7185	0.7185	0.7214	0.7208
ZIP	0.4503	0.4462	0.4462	0.4384	0.4461	0.4461	−0.0395	0.4504	0.4506	0.4462	0.4467	0.4467	−0.038	0.2166	0.6042	0.6042	0.6115	0.6115	0.6027	0.606
Loewe	0.178	0.1673	0.1673	0.1594	0.1742	0.1742	−0.0347	0.1769	0.1782	0.1677	0.1742	0.1742	−0.0329	0.3603	0.4502	0.4502	0.4527	0.4527	0.4518	0.4563
HSA	0.1773	0.1675	0.1675	0.1582	0.174	0.174	−0.0347	0.1778	0.1768	0.167	0.174	0.174	−0.0329	0.3559	0.4637	0.4637	0.4569	0.4569	0.4604	0.4611
Bliss	0.4503	0.4462	0.4462	0.4384	0.4461	0.4461	−0.0395	0.4504	0.4506	0.4462	0.4467	0.4467	−0.038	0.2166	0.6042	0.6042	0.6115	0.6115	0.6027	0.606
CSS	0.7999	0.7986	0.7986	0.7956	0.7988	0.7988	−0.031	0.7999	0.8	0.7987	0.7987	0.7987	−0.0313	0.6104	0.8478	0.8478	0.8459	0.8459	0.8471	0.8459
	Mathews																			
S	0.9018	0.8938	0.8066	0.8712	0.9235	0.9234	0.64	0.818	0.8934	0.9261	0.9283	0.9294	0.5083	0.6129	0.9028	0.927	0.9284	0.923	0.92	0.9287
ZIP	0.8858	0.8842	0.8551	0.8237	0.8894	0.8931	0.3739	0.7934	0.8302	0.891	0.8909	0.8946	0.1955	0.4186	0.8376	0.8933	0.9018	0.8883	0.8848	0.9022
Loewe	0.8753	0.8829	0.853	0.8281	0.8891	0.8876	0.2481	0.6977	0.7584	0.8909	0.8901	0.8901	0.1307	0.6391	0.8004	0.8799	0.8888	0.8721	0.8473	0.8882
HSA	0.6383	0.6464	0.5585	0.5008	0.646	0.6517	0.1429	0.4661	0.5703	0.6566	0.6515	0.6591	0.1667	0.3793	0.6403	0.682	0.6939	0.6528	0.6325	0.6826
Bliss	0.8434	0.845	0.8138	0.7472	0.8456	0.8495	0.3008	0.7379	0.7685	0.8491	0.8525	0.8532	0.2109	0.3207	0.7731	0.8646	0.8613	0.8557	0.8213	0.865
CSS	0.911	0.9158	0.8846	0.9088	0.9366	0.9358	0.4411	0.8261	0.8987	0.9378	0.9393	0.9397	0.1171	0.4796	0.9066	0.9372	0.9422	0.9375	0.9318	0.9412
	FORCINA_Mathews_O'Neil																			
S	0.9024	0.8986	0.8957	0.8862	0.9015	0.9058	0.5722	0.7765	0.8651	0.9017	0.9024	0.9067	0.8968	0.8752	0.915	0.9195	0.9192	0.919	0.917	0.9193
ZIP	0.6652	0.6295	0.6296	0.6089	0.6401	0.6723	0.2918	0.4135	0.5824	0.64	0.6462	0.6724	0.7034	0.6533	0.7151	0.7247	0.7259	0.7262	0.7209	0.7273
Loewe	0.8574	0.795	0.7983	0.8223	0.8427	0.8692	0.4107	0.602	0.7595	0.8456	0.8584	0.8685	0.7888	0.7746	0.8408	0.8671	0.8684	0.8646	0.8659	0.8739
HSA	0.5876	0.5531	0.5574	0.53	0.5688	0.5957	0.255	0.3112	0.5107	0.5644	0.5745	0.5954	0.6697	0.6418	0.6578	0.6594	0.6657	0.6656	0.6694	0.6651
Bliss	0.6177	0.5868	0.5919	0.5681	0.6016	0.627	0.2654	0.3729	0.5449	0.5957	0.6058	0.6253	0.6832	0.6444	0.6819	0.6908	0.6902	0.6939	0.6903	0.6936
CSS	0.9086	0.9039	0.9038	0.898	0.9079	0.9116	0.5048	0.7875	0.8734	0.9069	0.9086	0.9121	0.9037	0.8877	0.9191	0.9231	0.9232	0.9229	0.9228	0.9234
	ALMANAC_FORCINA_Mathews																			
S	0.7464	0.7397	0.737	0.7234	0.7729	0.7824	0.2573	0.4795	0.6062	0.7711	0.7887	0.7952	0.6584	0.6406	0.8032	0.8345	0.8337	0.8352	0.8308	0.8399
ZIP	0.6646	0.638	0.6383	0.6189	0.6524	0.6728	0.2344	0.5212	0.5838	0.6525	0.6594	0.6737	0.6809	0.647	0.7466	0.7697	0.7716	0.7713	0.7633	0.7717
Loewe	0.8353	0.6421	0.6419	0.7593	0.8092	0.8648	0.2859	0.4936	0.6958	0.813	0.8458	0.8685	0.7088	0.7006	0.8179	0.8632	0.8605	0.8593	0.85	0.8735
HSA	0.5222	0.4578	0.461	0.4247	0.4869	0.5344	0.1835	0.2763	0.4076	0.4931	0.5039	0.5356	0.6807	0.6614	0.691	0.7115	0.7107	0.7093	0.6955	0.7094
Bliss	0.579	0.5259	0.5272	0.4962	0.5491	0.5877	0.1829	0.3619	0.4658	0.5504	0.5606	0.5884	0.6655	0.6398	0.7212	0.7382	0.737	0.7365	0.7268	0.74
CSS	0.9196	0.9173	0.9168	0.9103	0.9261	0.9296	0.2732	0.8446	0.8743	0.9226	0.9278	0.9299	0.8772	0.8686	0.9303	0.9424	0.9423	0.9427	0.9413	0.9452
	ALMANAC_Mathews_O'Neil																			
S	0.8085	0.8026	0.7983	0.7796	0.8257	0.8335	0.3357	0.5999	0.6928	0.824	0.8357	0.8414	0.761	0.76	0.849	0.8692	0.8699	0.8698	0.8667	0.8731
ZIP	0.658	0.6211	0.6203	0.5731	0.6353	0.6646	0.1787	0.4098	0.5181	0.6317	0.6397	0.6646	0.698	0.6966	0.7461	0.7619	0.7616	0.7611	0.7517	0.762
Loewe	0.8292	0.6645	0.6673	0.7621	0.8055	0.8583	0.2689	0.5139	0.6861	0.8077	0.8392	0.8617	0.7201	0.7189	0.8168	0.8508	0.8529	0.8512	0.8461	0.8654
HSA	0.5809	0.533	0.5309	0.4849	0.5451	0.5868	0.2012	0.3248	0.4482	0.5494	0.5567	0.5887	0.7014	0.7018	0.717	0.7262	0.7191	0.721	0.7121	0.7189
Bliss	0.5613	0.5109	0.51	0.4578	0.5276	0.5686	0.1321	0.2688	0.4111	0.5267	0.5366	0.5681	0.6626	0.6636	0.6995	0.7141	0.712	0.7121	0.7044	0.7138
CSS	0.9326	0.931	0.9303	0.9232	0.9375	0.9405	0.3473	0.8624	0.89	0.9344	0.9384	0.9407	0.9063	0.9053	0.9419	0.9505	0.9502	0.9503	0.9494	0.9525
	ALMANAC_FORCINA_O'Neil																			
S	0.8067	0.8016	0.7966	0.7787	0.8241	0.8318	0.3393	0.5995	0.6929	0.8224	0.8339	0.8399	0.759	0.7561	0.8471	0.8678	0.8678	0.8681	0.8653	0.8713
ZIP	0.6545	0.6176	0.6176	0.5734	0.6321	0.6607	0.2133	0.4152	0.524	0.627	0.637	0.6616	0.6898	0.6817	0.7377	0.7537	0.7541	0.754	0.7455	0.7551
Loewe	0.829	0.6653	0.668	0.7625	0.806	0.8579	0.2794	0.5128	0.6872	0.807	0.8382	0.8614	0.7211	0.7203	0.8155	0.851	0.8525	0.85	0.8462	0.864
HSA	0.5805	0.5322	0.5315	0.4856	0.5485	0.5871	0.216	0.3287	0.4547	0.5492	0.5577	0.5888	0.697	0.6947	0.7112	0.7227	0.7174	0.7171	0.7095	0.7143
Bliss	0.5589	0.5108	0.512	0.4614	0.5309	0.5698	0.1656	0.2819	0.4223	0.5255	0.5388	0.5711	0.658	0.6538	0.6969	0.7098	0.7067	0.7091	0.7008	0.7105
CSS	0.9322	0.9303	0.9297	0.9228	0.9371	0.9401	0.347	0.8628	0.8903	0.9339	0.938	0.9403	0.9052	0.9031	0.9416	0.9499	0.9497	0.9496	0.949	0.952
	M1	M2	M3	M4	M5	M6	M7	M8	M9	M10	M11	M12	M13	M14	M15	M16	M17	M18	M19	M20

Supplementary Table 3. Performances (Pearson's r) of all models tested in this study in 1 vs. 1 inter-study cross-validation. The best performances for each train-test setting, each score, were marked as red. The features for all models are listed in **Supplementary Table 5.**

Train	Test	Score	M1	M2	M3	M4	M5	M6	M7	M8	M9	M10	M11	M12	M13	M14	M15	M16	M17	M18	M19	M20
ALMANAC	O'Neil	S	0.7034	0.7721	0.6561	0.8102	0.803	0.8052	0.3277	0.7297	0.7334	0.8142	0.8284	0.8284	0.023	0.0122	0.7138	0.7383	0.8095	0.7806	0.8273	0.8349
ALMANAC	O'Neil	ZIP	0.2765	0.2574	0.1867	0.2376	0.2407	0.1915	−0.0545	0.147	0.1125	0.2608	0.2492	0.1734	0.0828	0.064	0.1558	0.2653	0.2684	0.2244	0.2343	0.2591
ALMANAC	O'Neil	Loewe	0.4629	0.5142	0.4371	0.7459	0.7348	0.7424	0.1542	0.5438	0.5941	0.7058	0.7558	0.7598	0.1513	0.1555	0.488	0.5218	0.5853	0.5722	0.634	0.6281
ALMANAC	O'Neil	HSA	0.0939	0.1608	0.0579	0.1925	0.1664	0.0821	−0.0145	0.1875	0.0745	0.1579	0.1235	0.081	0.0472	0.0696	0.1348	0.165	0.154	0.1516	0.1553	0.1765
ALMANAC	O'Neil	Bliss	0.2352	0.2428	0.1973	0.2365	0.2322	0.2223	−0.0299	0.1933	0.126	0.2349	0.2228	0.2011	0.0922	0.0775	0.1257	0.2785	0.2836	0.2237	0.2251	0.2846
ALMANAC	O'Neil	CSS	0.6725	0.8384	0.8138	0.8369	0.8509	0.8359	0.2542	0.7424	0.7487	0.8435	0.8511	0.8392	0.1631	0.1679	0.7336	0.7324	0.8404	0.8263	0.8404	0.8579
ALMANAC	FORCINA	S	0.566	0.1449	0.2316	0.5254	0.5129	0.5147	0	0.5229	0.6063	0.5541	0.5311	0.5711	−0.0506	−0.0345	0.6251	0.597	0.6103	0.566	0.6161	0.5475
ALMANAC	FORCINA	ZIP	0.3164	0.3992	0.3887	0.3862	0.3933	0.4335		0.4056	0.4138	0.4494	0.4536	0.432	−0.01	−0.0084	0.4671	0.4625	0.4211	0.4499	0.4558	0.4285
ALMANAC	FORCINA	Loewe	0.0069	0.1689	0.1588	0.1122	0.1477	0.146	0	0.0929	0.1006	0.0534	0.1042	0.0948	0.0187	0.0309	0.1209	0.006	0.0663	0.0957	0.1025	0.1102
ALMANAC	FORCINA	HSA	−0.0074	0.1126	0.1274	0.0932	0.1216	0.0752	0	0.1013	0.0807	0.0735	0.1178	0.0883	−0.0381	−0.0399	0.095	0.0893	0.0873	0.0896	0.069	0.0836
ALMANAC	FORCINA	Bliss	0.3973	0.4588	0.4455	−0.0582	0.2205	0.3562		0.2261	0.3224	0.391	0.2932	0.2962	−0.0349	−0.0163	0.4688	0.4715	0.4317	0.45	0.4617	0.4322
ALMANAC	FORCINA	CSS	0.5171	0.7939	0.7735	0.7621	0.7689	0.7975	0	0.7405	0.7799	0.7412	0.7562	0.7807	0.1001	0.0989	0.8014	0.7517	0.7741	0.7657	0.797	0.7865
ALMANAC	Mathews	S	0.7485	0.6928	0.4333	0.799	0.8216	0.8331	0.4336	0.6494	0.6984	0.8467	0.8719	0.8686	−0.0602	−0.0464	0.7107	0.735	0.8381	0.792	0.8336	0.87
ALMANAC	Mathews	ZIP	0.785	0.6645	0.4054	0.69	0.641	0.6616	0.0226	0.6303	0.6154	0.6358	0.6655	0.6935	0.0649	0.0609	0.6403	0.7141	0.7287	0.6757	0.7123	0.6901
ALMANAC	Mathews	Loewe	0.4872	0.6994	0.7178	0.5646	0.6743	0.6107	0.0967	0.6927	0.6249	0.5335	0.6948	0.5932	−0.1017	−0.1235	0.7101	0.4885	0.402	0.7239	0.7478	0.4776
ALMANAC	Mathews	HSA	−0.0022	−0.1322	0.0548	−0.0217	0.1161	0.0202	0.0791	0.3745	0.3582	−0.1061	0.1448	−0.0309	−0.0044	−0.007	0.436	0.2965	0.4293	0.3481	0.3415	0.3531
ALMANAC	Mathews	Bliss	0.7186	0.694	0.3346	0.5691	0.5263	0.5261	−0.0295	0.5163	0.5128	0.6707	0.4889	0.5551	0.091	0.0978	0.6089	0.7671	0.7512	0.6648	0.7129	0.6898
ALMANAC	Mathews	CSS	0.5578	0.7878	0.7264	0.7248	0.8969	0.878	0.0485	0.7104	0.7272	0.8479	0.8974	0.8558	−0.1436	−0.1465	0.695	0.6935	0.8731	0.8433	0.8177	0.9053
O'Neil	ALMANAC	S	0.2818	0.4659	0.3928	0.5591	0.5575	0.4333	0.1091	0.3753	0.3666	0.5107	0.5728	0.4202	0.0317	0.0258	0.4076	0.3597	0.4984	0.5152	0.5904	0.6003
O'Neil	ALMANAC	ZIP	0.2869	0.3427	0.2677	0.3243	0.2967	0.1941	−0.0202	0.3517	0.323	0.3186	0.3077	0.209	0.0379	0.0473	0.3036	0.2811	0.323	0.3131	0.318	0.3486
O'Neil	ALMANAC	Loewe	−0.0687	0.5025	0.4338	0.6247	0.5944	0.2871	0.056	0.4254	0.524	0.6666	0.6709	0.3332	0.0942	0.0871	0.3989	0.0978	0.5362	0.5168	0.582	0.5723
O'Neil	ALMANAC	HSA	0.0394	0.2123	0.1179	0.1627	0.1459	0.133	0.0266	0.1769	0.1432	0.1968	0.1713	0.134	0.0159	0.0347	0.1661	0.1403	0.169	0.1425	0.1577	0.1594
O'Neil	ALMANAC	Bliss	0.1913	0.2099	0.0316	0.1815	0.1349	0.141	−0.0116	0.2208	0.1977	0.203	0.187	0.1553	0.032	0.0338	0.1635	0.2402	0.2405	0.2296	0.1983	0.2694
O'Neil	ALMANAC	CSS	0.2676	0.8812	0.8577	0.8669	0.8802	0.4692	0.1286	0.8146	0.8088	0.8823	0.8839	0.476	0.1005	0.0995	0.795	0.3719	0.8779	0.8707	0.8763	0.886
O'Neil	FORCINA	S	0.4837	−0.4228	−0.4979	0.5472	0.0088	0.0775		0.4379	0.5287	−0.4143	0.5367	0.1703	0.0278	0.0088	0.4807	0.3998	−0.4137	0.5405	0.5839	0.5575
O'Neil	FORCINA	ZIP	0.1022	0.1743	0.1699	0.3747	−0.1659	−0.2152	0	0.4407	0.4168	0.1891	−0.0159	0.1291	0.0136	0.0278	0.451	0.3453	0.4112	0.442	0.4532	0.3745
O'Neil	FORCINA	Loewe	−0.121	0.0117	0.1483	0.1548	0.1484	0.098	0	0.1066	0.1097	0.1335	0.1562	0.0972	0.0233	0.0124	−0.0379	0.0824	0.0661	0.0436	0.144	0.1403
O'Neil	FORCINA	HSA	0.1388	0.0766	0.085	0.1102	0.0947	0.1254	0	0.1412	0.1417	0.0663	0.0642	0.1421	0.0452	0.0432	0.1709	0.0088	0.1832	0.1144	0.1461	0.1464
O'Neil	FORCINA	Bliss	0.2867	−0.2602	0.1662	−0.0842	−0.2438	−0.3018	0	0.3777	0.4316	−0.3174	−0.3978	0.041	0.0306	0.0273	0.2048	0.3127	0.0517	0.3697	0.3098	0.3667
O'Neil	FORCINA	CSS	0.3831	0.7948	0.783	0.7711	0.7766	0.5763	0	0.7387	0.7913	0.7696	0.7869	0.4692	0.1197	0.1178	0.7664	0.7117	0.8036	0.7961	0.7653	0.7775
O'Neil	Mathews	S	0.6031	0.7899	0.1961	0.7848	0.7689	0.7555	0.3114	0.6133	0.5672	0.8168	0.7901	0.7259	0.1908	0.1396	0.7284	0.6964	0.7953	0.704	0.8212	0.8399
O'Neil	Mathews	ZIP	0.6959	0.5616	0.2523	0.5702	0.5682	0.5567	0.0371	0.5718	0.5825	0.6597	0.4472	0.643	0.1182	0.1428	0.6232	0.6518	0.6222	0.4758	0.4892	0.624
O'Neil	Mathews	Loewe	0.4762	0.6692	0.4325	0.5971	0.5172	0.3994	0.0238	0.6188	0.5896	0.7454	0.5017	0.4627	0.0222	0.0542	0.6042	0.5581	0.7624	0.6348	0.5172	0.6029
O'Neil	Mathews	HSA	0.389	0.0596	0.0783	0.0481	0.0937	0.1741	−0.0274	0.327	0.3371	0.1639	0.0726	0.119	0.1032	0.0863	0.4274	0.3044	0.2709	0.1126	0.1515	0.0529
O'Neil	Mathews	Bliss	0.3746	0.4699	−0.105	0.2949	0.1568	0.1243	0.0711	0.4261	0.4304	0.4973	0.0637	0.2113	0.1783	0.1657	0.4663	0.6427	0.5203	0.3271	0.2183	0.4367
O'Neil	Mathews	CSS	0.5677	0.8604	0.6666	0.7261	0.8281	0.6171	−0.0219	0.6921	0.687	0.8586	0.8308	0.5784	0.0887	0.0798	0.7766	0.5911	0.8826	0.8111	0.8329	0.8731
FORCINA	ALMANAC	S	0.0846	0.0759	0.0574	0.2092	0.0685	−0.0736	0.0465	0.142	0.1444	0.0396	0.0198	0.0198		0.0347	0.2041	0.2041	0.1183	0.1001	0.1584	0.1268
FORCINA	ALMANAC	ZIP	0.1377	0.1547	0.1499	0.1283	0.1678	−0.0912	−0.0326	0.182	0.1835	−0.0673	−0.0832	−0.0832	0	0.0648	0.2176	0.2176	0.2061	0.2002	0.2024	0.1976
FORCINA	ALMANAC	Loewe	0.0051	−0.0146	−0.0027	0.1078	−0.0421	0.0275	0.0243	−0.0609	−0.0587	−0.005	0.038	0.038		0.0952	0.0646	0.0646	0.0625	0.0626	0.0617	0.0741
FORCINA	ALMANAC	HSA	0.0064	−0.0082	−0.0248	−0.0781	−0.0477	0.091	−0.0224	0.0329	0.0344	0.0851	0.0899	0.0899	0	0.0524	0.1065	0.1065	0.0917	0.0917	0.1061	0.0847
FORCINA	ALMANAC	Bliss	0.1582	0.1996	0.1938	0.138	0.2131	−0.1747	−0.0023	0.1101	0.1129	−0.1787	−0.1737	−0.1737	0	0.0722	0.1964	0.1964	0.2345	0.2273	0.1876	0.219
FORCINA	ALMANAC	CSS	0.1412	0.253	0.2386	0.1834	0.2614	0.3449	−0.0441	0.6511	0.6501	0.4219	0.445	0.445	0	0.2224	0.5022	0.5022	0.4379	0.4296	0.4288	0.5072
FORCINA	O'Neil	S	0.2738	0.3086	0.2666	0.2991	0.2758	−0.2887	−0.0077	0.149	0.1527	0.0131	−0.025	−0.025	0	0.1221	0.2579	0.2579	0.2569	0.2323	0.1579	0.2262
FORCINA	O'Neil	ZIP	0.1445	0.2059	0.193	0.0647	0.2041	−0.1598	0.0011	0.0293	0.0316	−0.1264	−0.1352	−0.1352	0	−0.006	0.0756	0.0756	0.1441	0.1454	0.0669	0.1283
FORCINA	O'Neil	Loewe	0.051	−0.0445	−0.0529	−0.1323	−0.0945	0.2444	−0.0679	0.0318	0.0366	0.1554	0.2276	0.2276	0	0.0723	0.0873	0.0873	0.0634	0.0644	0.0893	0.0665
FORCINA	O'Neil	HSA	0.0382	0.011	−0.0192	−0.0544	−0.0268	0.0871	−0.0445	0.0304	0.0287	0.063	0.0791	0.0791	0	0.0267	0.0777	0.0777	0.047	0.0462	0.0682	0.0374
FORCINA	O'Neil	Bliss	0.1393	0.218	0.2006	0.1131	0.208	−0.2075	0.0087	−0.0023	0.0026	−0.1864	−0.1744	−0.1744	0	0.0126	0.0657	0.0657	0.1336	0.1335	0.0591	0.1188
FORCINA	O'Neil	CSS	0.2552	0.1802	0.1618	0.2955	0.1929	0.2968	−0.0872	0.5667	0.5657	0.3789	0.3978	0.3978	0	0.3331	0.5135	0.5135	0.406	0.395	0.4667	0.4697
FORCINA	Mathews	S	0.4269	0.4979	0.4813	0.5746	0.5644	−0.1097	0.0537	0.4944	0.4988	−0.1697	−0.1101	−0.1101	0	0.2066	0.5699	0.5699	0.438	0.416	0.4706	0.2989
FORCINA	Mathews	ZIP	0.6413	0.5308	0.4581	0.5014	0.5376	−0.2575	−0.0186	0.5144	0.5104	−0.2313	−0.2108	−0.2108	0	0.2081	0.433	0.433	0.5234	0.5143	0.4584	0.4658
FORCINA	Mathews	Loewe	−0.0224	−0.1323	−0.1122	−0.1945	−0.2389	0.3173	−0.0391	0.0052	0.0081	0.3137	0.2617	0.2617		−0.111	0.1004	0.1004	−0.0331	−0.0296	0.1104	−0.0395
FORCINA	Mathews	HSA	0.1439	−0.0061	−0.0327	−0.1851	0.0476	0.1489	−0.0323	0.0916	0.0863	0.1334	0.1537	0.1537	0	−0.104	0.1152	0.1152	0.1028	0.0964	0.1273	0.14
FORCINA	Mathews	Bliss	0.6072	0.4847	0.453	0.467	0.4744	−0.2234	−0.0034	0.4274	0.4236	−0.266	−0.2508	−0.2508	0	0.19	0.3837	0.3837	0.4728	0.4672	0.4035	0.4113
FORCINA	Mathews	CSS	0.4221	−0.0593	−0.0182	0.4442	−0.067	−0.066	−0.0401	0.5689	0.5682	0.0289	0.0572	0.0572	0	0.2604	0.5333	0.5333	0.188	0.2141	0.4818	0.2635
Mathews	ALMANAC	S	0.1877	0.3506	0.2066	0.3965	0.5009	0.4702	0.0978	0.2555	0.2621	0.3113	0.473	0.4369	0	−0.0236	0.2772	0.3078	0.3174	0.3388	0.4401	0.4668
Mathews	ALMANAC	ZIP	0.1574	0.1747	0.1145	0.3093	0.1656	0.161	0.0352	0.2423	0.063	0.1244	0.0829	0.1277	0	0.0452	0.2223	0.2944	0.2165	0.2063	0.2797	0.2211
Mathews	ALMANAC	Loewe	−0.0175	0.271	0.2683	0.2138	0.2427	0.2234	0.0271	0.1913	0.1819	0.2394	0.2211	0.2096	0	−0.0801	0.1699	0.1286	0.2169	0.2024	0.159	0.1958
Mathews	ALMANAC	HSA	−0.0109	0.1475	0.1793	0.1041	0.1628	0.131	0.0165	0.092	0.1107	0.1454	0.1551	0.12	0	0.0498	0.094	0.0081	0.1915	0.1802	0.1558	0.1868
Mathews	ALMANAC	Bliss	0.2326	0.1545	0.1472	0.1243	0.1378	0.1935	0.0146	0.1903	0.1552	0.1646	0.1434	0.1852		0.0107	0.2134	0.2832	0.2396	0.2744	0.2336	0.2519
Mathews	ALMANAC	CSS	−0.1503	0.8333	0.8335	0.6378	0.8478	0.7817	−0.0171	0.6025	0.4511	0.8277	0.831	0.7761	0	0.107	0.5474	0.6752	0.8233	0.8229	0.7559	0.8183
Mathews	O'Neil	S	0.6361	0.7441	0.6788	0.715	0.7558	0.778	0.3001	0.6939	0.6715	0.7093	0.7677	0.7776	0.1356	0.1565	0.6743	0.6935	0.7164	0.7165	0.7675	0.7733
Mathews	O'Neil	ZIP	0.0641	−0.0537	0.0518	0.0439	−0.0289	−0.0092	−0.015	0.0982	0.0663	−0.0313	−0.0409	−0.0221	0.0638	0.0355	0.1012	0.177	0.1339	0.1636	0.1236	0.1493
Mathews	O'Neil	Loewe	0.3769	0.5527	0.564	0.5019	0.5544	0.5383	−0.0429	0.4258	0.3837	0.5496	0.5472	0.5281	0	0.1233	0.3983	0.4695	0.5552	0.5436	0.506	0.547
Mathews	O'Neil	HSA	0.2078	0.1972	0.2268	0.1599	0.21	0.2172	−0.0367	0.1276	0.1254	0.1991	0.2086	0.2063	−0.0295	0.11	0.1768	0.2366	0.215	0.1885	0.2062	0.216
Mathews	O'Neil	Bliss	0.0866	−0.0353	0.0656	−0.0038	−0.0568	0.0384	0.0025	0.1108	0.1301	−0.0299	−0.044	0.01	0.0686	0.0851	0.1813	0.1872	0.1502	0.2029	0.1408	0.116
Mathews	O'Neil	CSS	0.4416	0.7645	0.792	0.6471	0.78	0.7601	−0.0867	0.6579	0.5386	0.7652	0.7734	0.7493	0.1356	0.1996	0.5285	0.6609	0.7838	0.7869	0.7216	0.7824
Mathews	FORCINA	S	0.4522	−0.4267	−0.3899	0.6324	0.5313	0.5394		0.4828	0.5515	0.2437	0.5638	0.5981	0.0095	0.1311	0.5647	0.4388	0.3931	0.4866	0.6323	0.626
Mathews	FORCINA	ZIP	0.4545	0.4508	0.2807	0.4522	0.3691	0.4161	0	0.4021	0.4178	0.1387	0.2924	0.4101	0.0229	0.0885	0.4547	0.4547	0.0067	0.0942	0.4448	0.2624
Mathews	FORCINA	Loewe	0.0824	0.1195	0.1323	0.1363	0.1304	0.1256	0	0.0876	0.0761	0.1095	0.1187	0.1091	−0.0268	−0.0221	0.0952	0.0753	0.1108	0.1162	0.0991	0.1301
Mathews	FORCINA	HSA	0.0961	0.1193	0.1424	0.1638	0.1042	0.1153		0.1201	0.1321	0.1102	0.0935	0.1035	−0.0027	−0.0581	0.0899	0.0636	0.0745	0.1344	0.1452	0.0777
Mathews	FORCINA	Bliss	0.4317	−0.1625	−0.2661	0.4446	0.0261	0.1767	0	0.361	0.4379	0.0164	0.1932	0.2288	0.0216	0.0989	0.4179	0.4346	−0.0026	0.0812	0.4196	0.0156
Mathews	FORCINA	CSS	0.438	0.6462	0.624	0.6456	0.6527	0.6899		0.6422	0.7514	0.6733	0.643	0.6352	−0.0235	0.1756	0.7394	0.6291	0.6395	0.7238	0.7483	0.6154

Supplementary Table 4. Performances (Pearson's r) of all models tested in this study in 3 vs. 1 inter-study cross-validation. The best performances for each train-test setting, each score, were marked as red. The features for all models are listed in **Supplementary Table 5.**

Train: FORCINA_Mathews_O'Neil — **Test:** ALMANAC

	M1	M2	M3	M4	M5	M6	M7	M8	M9	M10	M11	M12	M13	M14	M15	M16	M17	M18	M19	M20
S	0.2997	0.4638	0.4707	0.5584	0.582	0.5668	0.1135	0.3875	0.3689	0.5389	0.6035	0.5768	0.0381	0.0167	0.4007	0.3311	0.4942	0.5055	0.5595	0.5614
ZIP	0.2845	0.3556	0.343	0.3356	0.3327	0.282	−0.0114	0.3646	0.34	0.3463	0.3419	0.2625	0.0361	0.0127	0.1863	0.1182	0.2525	0.217	0.1804	0.1751
Loewe	−0.1386	0.5231	0.4892	0.6326	0.6458	0.6623	0.049	0.4266	0.5186	0.6658	0.69	0.6756	0.0957	0.0095	0.3719	0.1885	0.5299	0.5115	0.5816	0.5959
HSA	0.0054	0.2189	0.1633	0.1408	0.2129	0.1325	0.0223	0.1571	0.1377	0.2071	0.2113	0.1491	0.0115	0.0056	0.0853	0.0456	0.1143	0.0947	0.088	0.1104
Bliss	0.2114	0.2316	0.2196	0.2069	0.2791	0.2791	−0.0136	0.2421	0.2157	0.2611	0.2671	0.2217	0.0344	0.0336	0.1639	0.233	0.1937	0.1872	0.1798	0.1953
CSS	0.2465	0.8803	0.8705	0.8671	0.8918	0.8735	0.1275	0.8157	0.8007	0.882	0.8922	0.8706	0.0992	0.2102	0.7755	0.6486	0.8645	0.867	0.8659	0.8832

Train: ALMANAC_FORCINA_Mathews — **Test:** O'Neil

	M1	M2	M3	M4	M5	M6	M7	M8	M9	M10	M11	M12	M13	M14	M15	M16	M17	M18	M19	M20
S	0.6791	0.7738	0.6255	0.8077	0.8019	0.8134	0.327	0.7333	0.7338	0.8227	0.8283	0.8297	0.0203	0.0032	0.6958	0.7284	0.8116	0.7905	0.8193	0.8328
ZIP	0.2367	0.2725	0.1916	0.2341	0.2341	0.2137	−0.0484	0.1563	0.1213	0.2657	0.2424	0.2273	0.0061	−0.042	0.0319	0.1366	0.1618	0.1207	0.1154	0.1333
Loewe	0.5188	0.5448	0.5178	0.746	0.749	0.7612	0.1394	0.5406	0.5977	0.7239	0.7634	0.7758	0.136	0.1743	0.4629	0.5119	0.5741	0.5533	0.6315	0.6335
HSA	0.1426	0.1643	0.099	0.1895	0.1863	0.1053	0.0173	0.184	0.1006	0.1446	0.1614	0.14	0.0779	0.0461	0.1081	0.1628	0.1146	0.1146	0.1143	0.123
Bliss	0.2428	0.2309	0.1546	0.2306	0.2383	0.2336	−0.0278	0.1912	0.1346	0.2341	0.2277	0.2304	0.0434	0.0158	0.0314	0.1933	0.1764	0.1389	0.0963	0.2083
CSS	0.6885	0.8419	0.8184	0.8374	0.8585	0.8465	0.265	0.7438	0.7484	0.843	0.854	0.8518	0.1674	0.2874	0.7256	0.734	0.8385	0.8314	0.8317	0.8555

Train: ALMANAC_Mathews_O'Neil — **Test:** FORCINA

	M1	M2	M3	M4	M5	M6	M7	M8	M9	M10	M11	M12	M13	M14	M15	M16	M17	M18	M19	M20
S	0.579	−0.1093	−0.2428	0.512	0.5187	0.2213	0	0.5357	0.5987	0.5262	0.5473	0.5623	−0.0331	0.0124	0.6003	0.5665	0.6079	0.5707	0.593	0.5487
ZIP	0.4066	0.3845	0.3801	0.3553	0.3555	0.4704		0.419	0.4457	0.4359	0.4466	0.4696	0.0344	0.0583	0.4667	0.4704	0.4548	0.4535	0.4728	0.4663
Loewe	−0.0083	0.1712	0.1622	0.1305	0.1712	0.1252	0	0.1052	0.1109	0.1134	0.1648	0.1411	0.0092	−0.0242	0.0975	0.0165	0.1115	0.1372	0.1464	0.1459
HSA	0.0402	0.1175	0.1129	0.1121	0.1365	0.1794	0	0.1316	0.1249	0.0868	0.1254	0.0886	0.0304	0.0111	0.1188	0.1119	0.0972	0.0927	0.1154	0.1012
Bliss	0.3919	0.4546	0.446	−0.056	0.3246	−0.1195	0	0.4584	0.4274	0.2895	0.4115	0.4482	0.0265	0.0571	0.4519	0.3835	0.4322	0.4511	0.4556	0.4328
CSS	−0.2484	0.7893	0.78	0.7723	0.7885	0.7746	0	0.7484	0.7924	0.7296	0.7315	0.758	0.1625	0.1715	0.7882	0.716	0.7581	0.7819	0.7894	0.7752

Train: ALMANAC_FORCINA_O'Neil — **Test:** Mathews

	M1	M2	M3	M4	M5	M6	M7	M8	M9	M10	M11	M12	M13	M14	M15	M16	M17	M18	M19	M20
S	0.7225	0.7719	0.5404	0.8007	0.8672	0.8799	0.4347	0.6708	0.7061	0.8541	0.8669	0.8749	−1e−04	−0.3197	0.6143	0.6756	0.8471	0.8238	0.8398	0.8889
ZIP	0.8249	0.6433	0.5666	0.691	0.6508	0.7363	0.0198	0.6351	0.6263	0.6653	0.6778	0.7479	0.183	−0.0612	0.5315	0.7018	0.6414	0.602	0.6359	0.6712
Loewe	0.5858	0.7249	0.6705	0.614	0.7012	0.7036	0.0902	0.6662	0.6425	0.7896	0.7266	0.6665	−0.1099	−0.0093	0.6784	0.6819	0.7567	0.6882	0.7164	0.741
HSA	0.3641	0.0354	0.0645	0.0134	0.0853	0.2315	0.0785	0.4105	0.4303	0.0068	0.0351	0.1826	0.0157	0.1717	0.4222	0.3174	0.4012	0.3077	0.2784	0.3692
Bliss	0.7622	0.6021	0.4286	0.5778	0.5985	0.6569	0.0384	0.5265	0.5397	0.6328	0.5947	0.6599	0.1636	−0.0944	0.473	0.7193	0.5895	0.5274	0.6838	0.5797
CSS	0.5521	0.8443	0.8114	0.7596	0.8807	0.8987	−0.0406	0.7185	0.7323	0.8716	0.886	0.9012	0.0651	0.0273	0.6546	0.6626	0.8751	0.8707	0.8155	0.917

Supplementary Table 5. Table legend for all models in Supplementary Table 2-4.

model	feature
**M1**	drc_baseline
**M2**	drc_intp_linear
**M3**	drc_intp_lagrange
**M4**	drc_intp_4PL
**M5**	drc_intp_linear+drc_intp_lagrange+drc_intp_4PL
**M6**	drc_baseline+drc_intp_linear+drc_intp_lagrange+drc_intp_4PL
**M7**	monotherapy_ic50
**M8**	monotherapy_ri
**M9**	monotherapy_ri+monotherapy_ic50
**M10**	monotherapy_ic50+monotherapy_ri+drc_intp_linear
**M11**	monotherapy_ic50+monotherapy_ri+drc_intp_linear+drc_intp_lagrange+drc_intp_4PL
**M12**	monotherapy_ic50+monotherapy_ri+drc_baseline+drc_intp_linear+drc_intp_lagrange+drc_intp_4PL
**M13**	drug_categorical+cell_line_categorical
**M14**	drug_categorical+cell_line_categorical+cancer_gene_expression+chemical_structure
**M15**	drug_categorical+cell_line_categorical+cancer_gene_expression+chemical_structure+monotherapy_ic50+monotherapy_r
**M16**	drug_categorical+cell_line_categorical+cancer_gene_expression+chemical_structure+monotherapy_ic50+monotherapy_ri+drc_baseline
**M17**	drug_categorical+cell_line_categorical+cancer_gene_expression+chemical_structure+monotherapy_ic50+monotherapy_ri+drc_intp_linear
**M18**	drug_categorical+cell_line_categorical+cancer_gene_expression+chemical_structure+monotherapy_ic50+monotherapy_ri+drc_intp_lagrange
**M19**	drug_categorical+cell_line_categorical+cancer_gene_expression+chemical_structure+monotherapy_ic50+monotherapy_ri+drc_intp_4PL
**M20**	drug_categorical+cell_line_categorical+cancer_gene_expression+chemical_structure+monotherapy_ic50+monotherapy_ri+drc_intp_linear+drc_intp_lagrange+drc_intp_4PL

Supplementary Table 6. Example of the drug combination dataset provided by DrugComb. For each combination, two drugs (drug_row and drug_col) and the treated cell line (cell_line_name) is shown. Five synergy scores (ZIP, Bliss, Loewe, HSA and S) and sensitivity score (css) are shown for each experiment. For each drug combination, there could be more than one replicated experiment. And the source of each experiment can be traced using the block id.

block id	drug_row	drug_col	cell_line_name	CSS	ZIP	Bliss	Loewe	HSA	S
1	5-FU	ABT-888	A2058	30.869	3.865	6.256	-2.951	5.537	19.839
2	5-FU	ABT-888	A2058	27.46	8.247	12.334	3.126	11.614	16.43
3	5-FU	ABT-888	A2058	29.901	6.063	11.660	2.452	10.941	18.871
4	5-FU	ABT-888	A2058	24.016	-4.280	5.145	-4.063	4.426	12.986

Supplementary figures

Supplementary Figure 1. Reproducibility of the six drug combination activity measurements within and between studies used in this dataset. The reproducibility is measured by Pearson's correlation between the response score (S, CSS, Bliss, HSA, Loewe, and ZIP) of the same treatment-cell line combinations. The reproducibility can be used as a standard to evaluate the prediction performance in study and cross study. For cross-study, only studies with overlapped treatment-cell line combinations were evaluated. FORCINA is not included in this figure since there are neither replicates within this dataset nor between this dataset and the others.

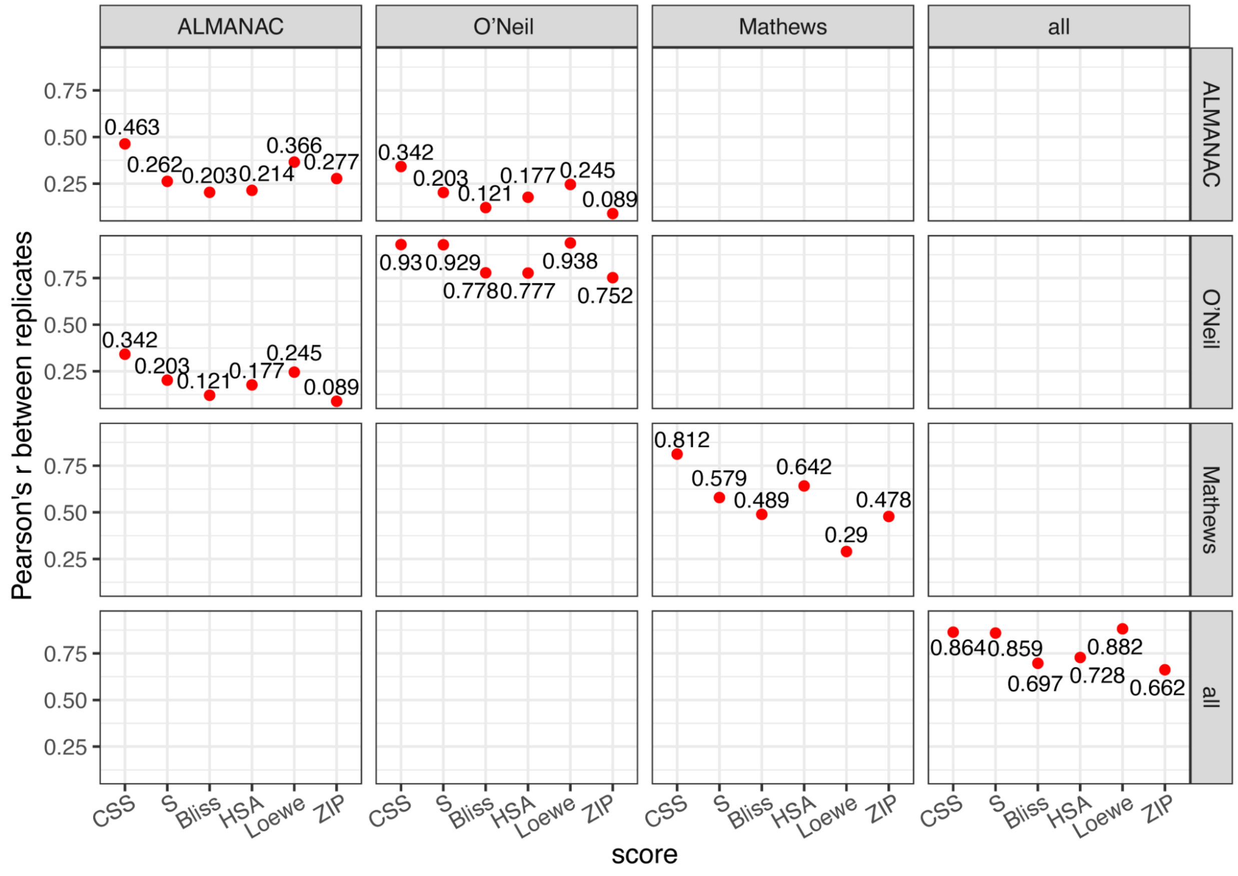

Supplementary Figure 2. Histograms show the concentration ranges (log10) for single drug dose-response measurement adopted in the four high-throughput screening studies. Different colors (red, purple, blue and green) denote the HTS study (ALMANAC, O'Neil, Mathews and FORCINA) the monotherapy dose setting is used for each dose-response curve. Since all doses start from 0, the log10 of the first concentration (-∞) is not shown in this graph.

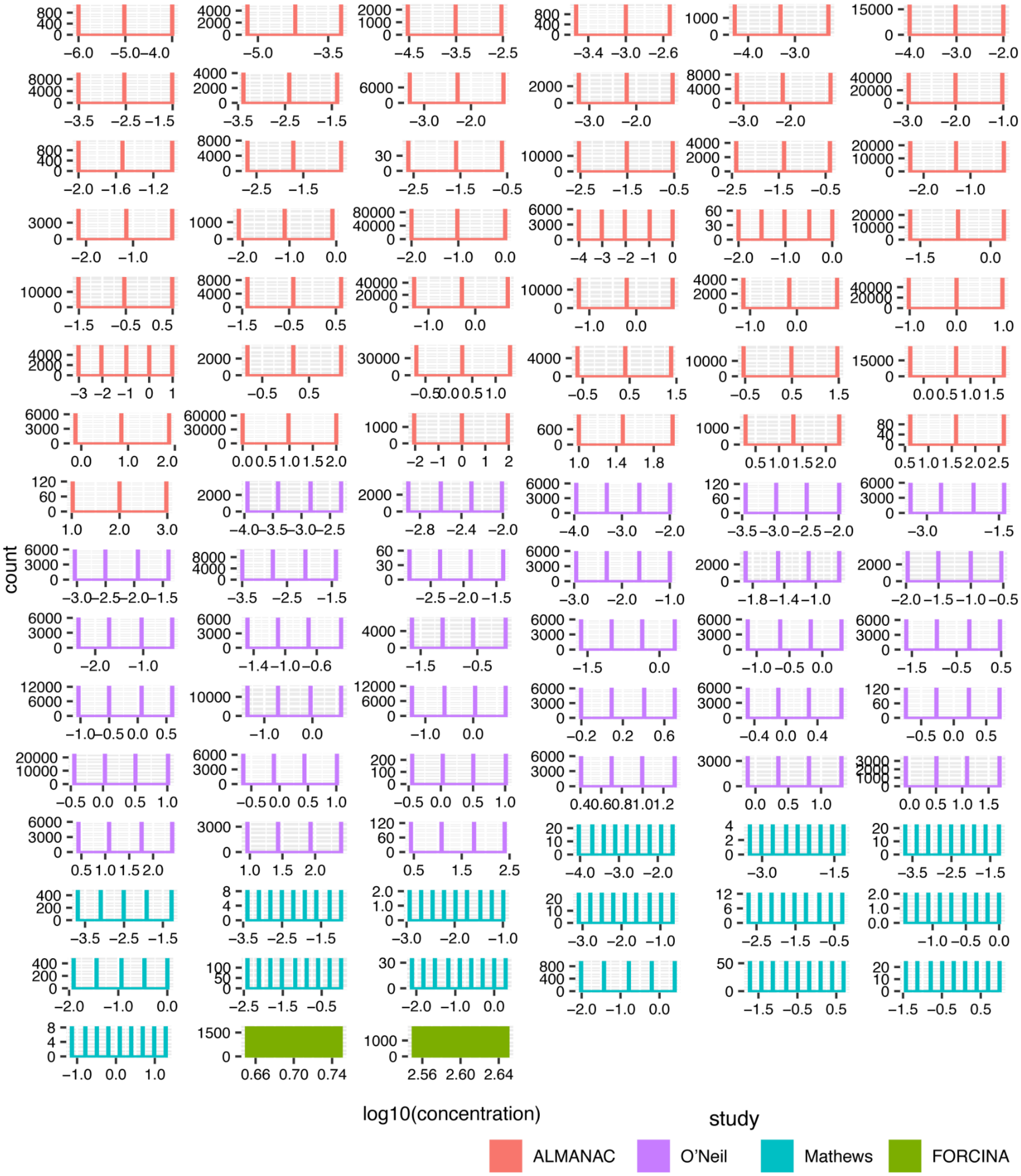

Supplementary Figure 3. Reproducibility of monotherapy response measurements in different studies used in this dataset. The reproducibility is measured by Pearson's correlation between the response score (IC50 and RI (relative inhibition)), and statistics of the dose-response curve (min, mean, median, and max of inhibition) of the same monotherapy treatment-cell line combinations. The reproducibility can be used as a reference for the prediction performance in-study and cross-study. For cross-study, only studies with overlapped monotherapy treatment-cell line combinations were evaluated. FORCINA is not included in this figure since there are no replicates in this dataset. “all” refers to the overall replicability across all studies.

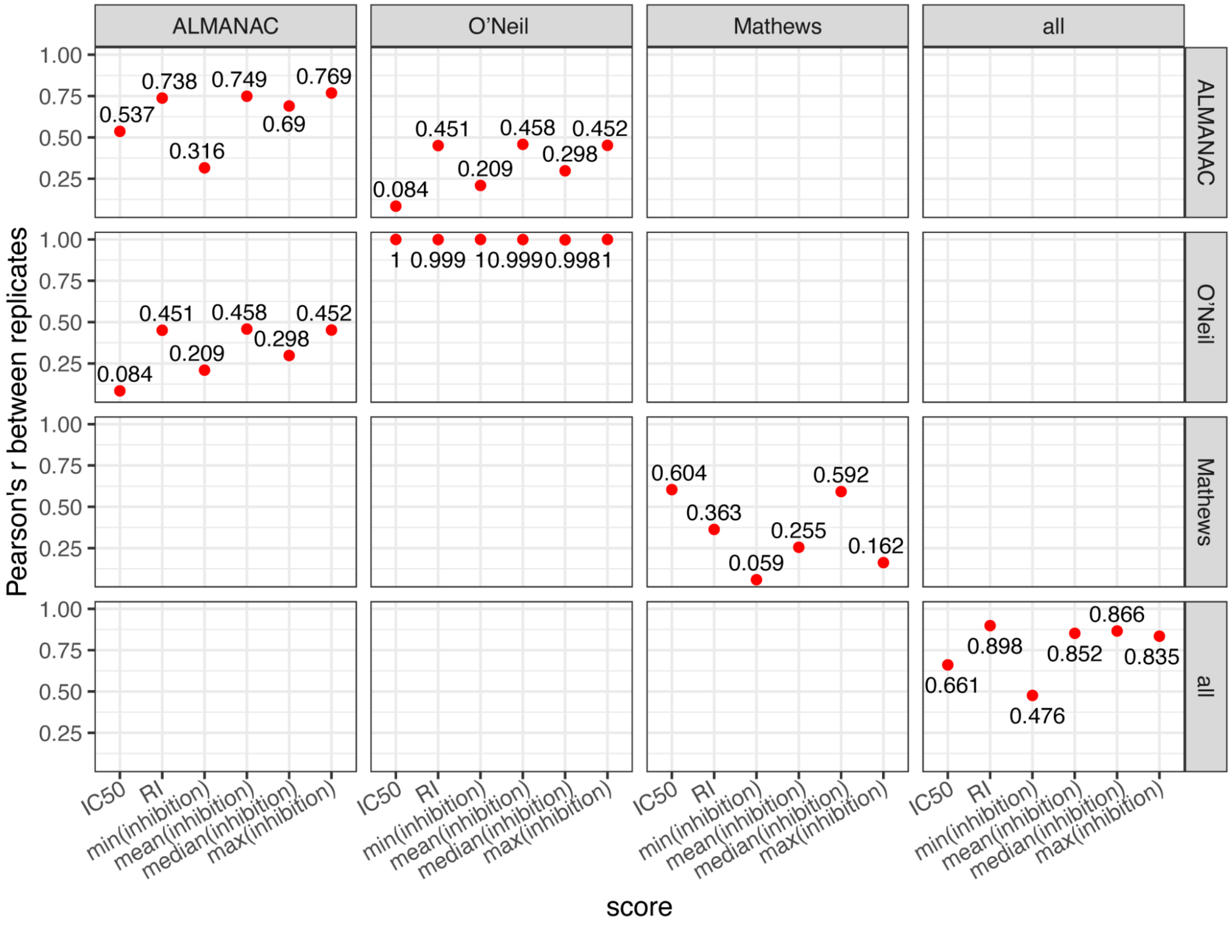

Supplementary Figure 4. Pair-wise comparison of model performances over each combination treatment response score (CSS, Bliss, HSA, Loewe, ZIP, S) using p-values from paired t-test and performance ratios (PR). The models in this figure are corresponding to Figure 2 D. A. intra-study cross-validation. B. inter-study cross-validation.

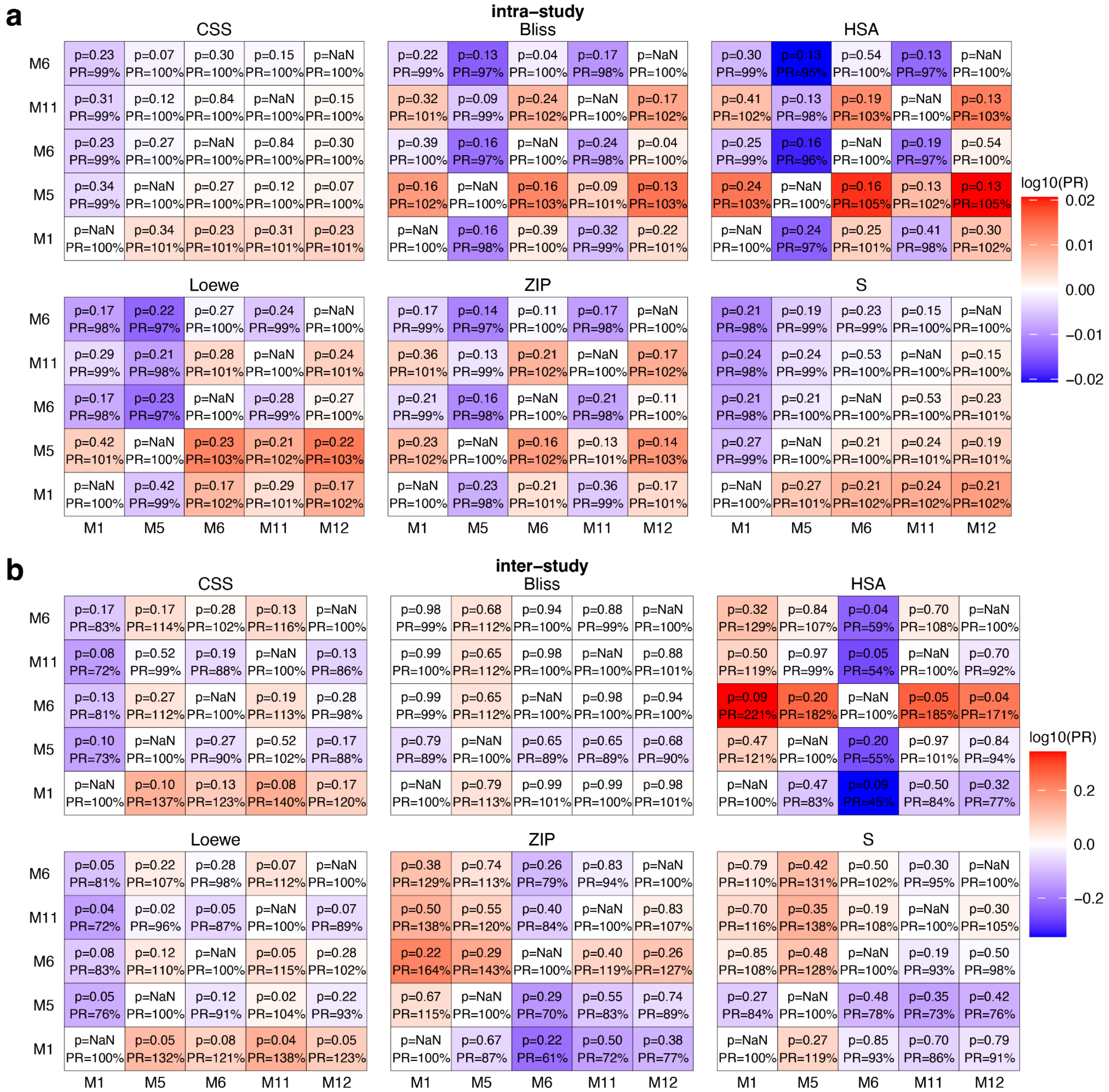

Supplementary Figure 5. The comparison between different interpolation methods for dose-response curves. A. example of original drc and different interpolation methods. The original dose-response curve contains only five doses, and is interpolated to the maximum length, which is ten doses. Also, for the interpolation models, we used the magnitude of interpolated inhibition as the final features. B. Performances of all interpolation models in different training (horizontal) and testing (vertical) settings. C. Comparison of performances between models by paired t-test and performance ratio (PR) on the average.

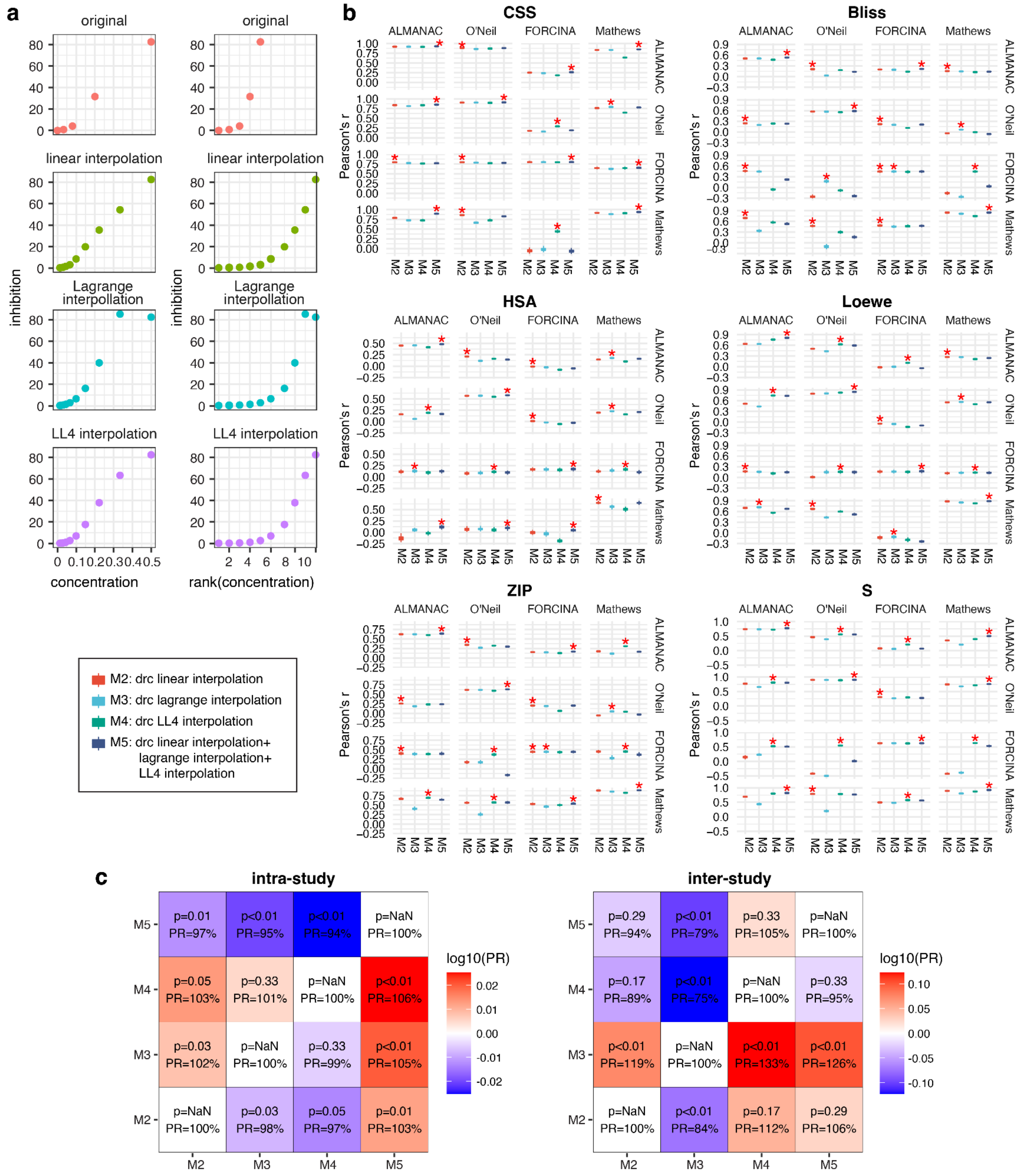

Supplementary Figure 6. Pair-wise comparison of model performances over each combination treatment response score (CSS, Bliss, HSA, Loewe, ZIP, S) using p-values from paired t-test and performance ratios (PR). The models in this figure are corresponding to Supplementary Figure 5 C. A. intra-study cross-validation. B. inter-study cross-validation.

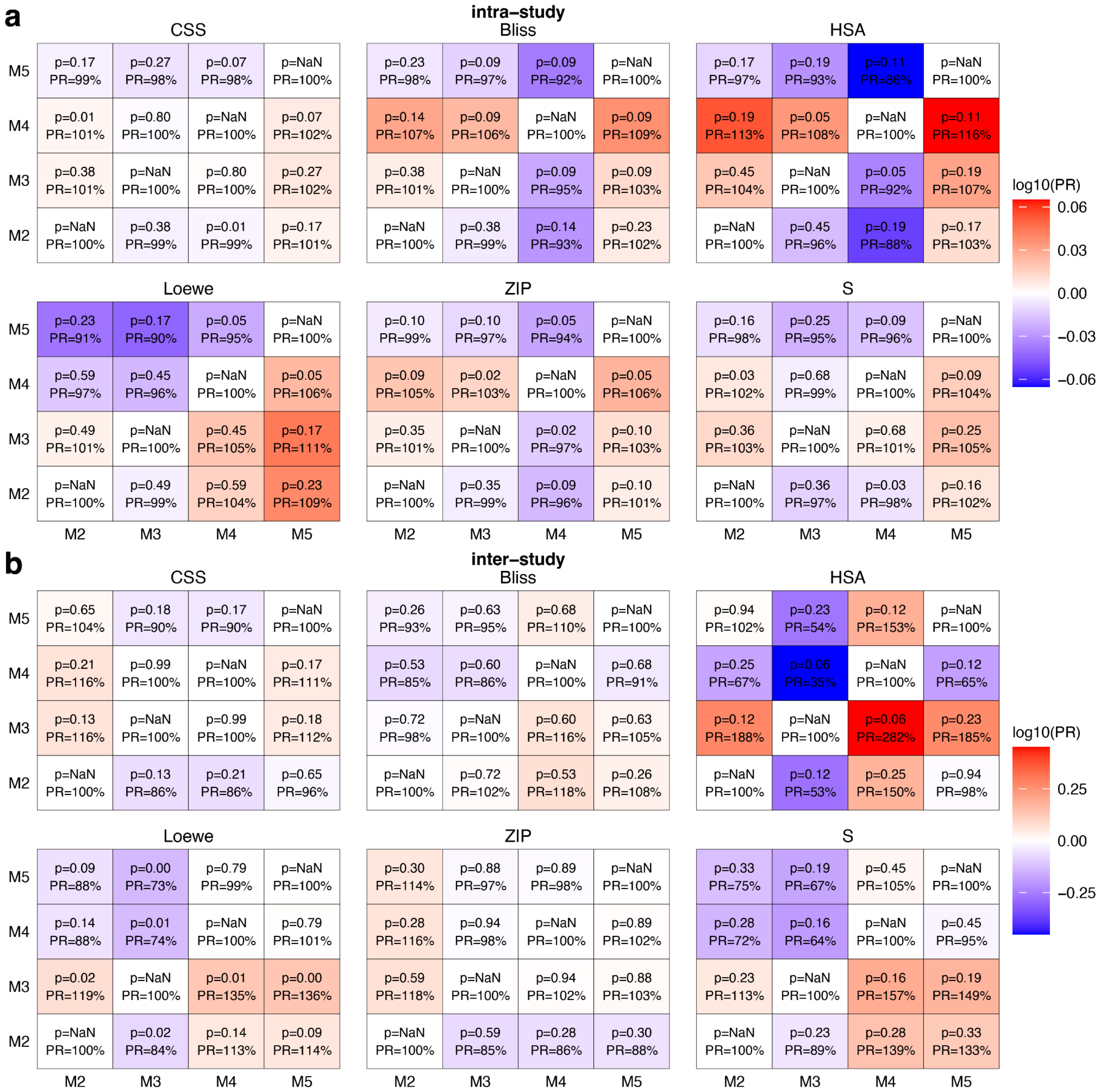

Supplementary Figure 7. The comparison between using different monotherapy efficacy scores as features. Both IC50 and RI (relative inhibition) are used to measure the monotherapy efficacy. We tested the performances by using either or both in intra-and inter-study predictions. A. Performances of all interpolation models in different training (top) and testing (right) settings. C. Comparison of performances between models by paired t-test and performance ratio (PR) on the average.

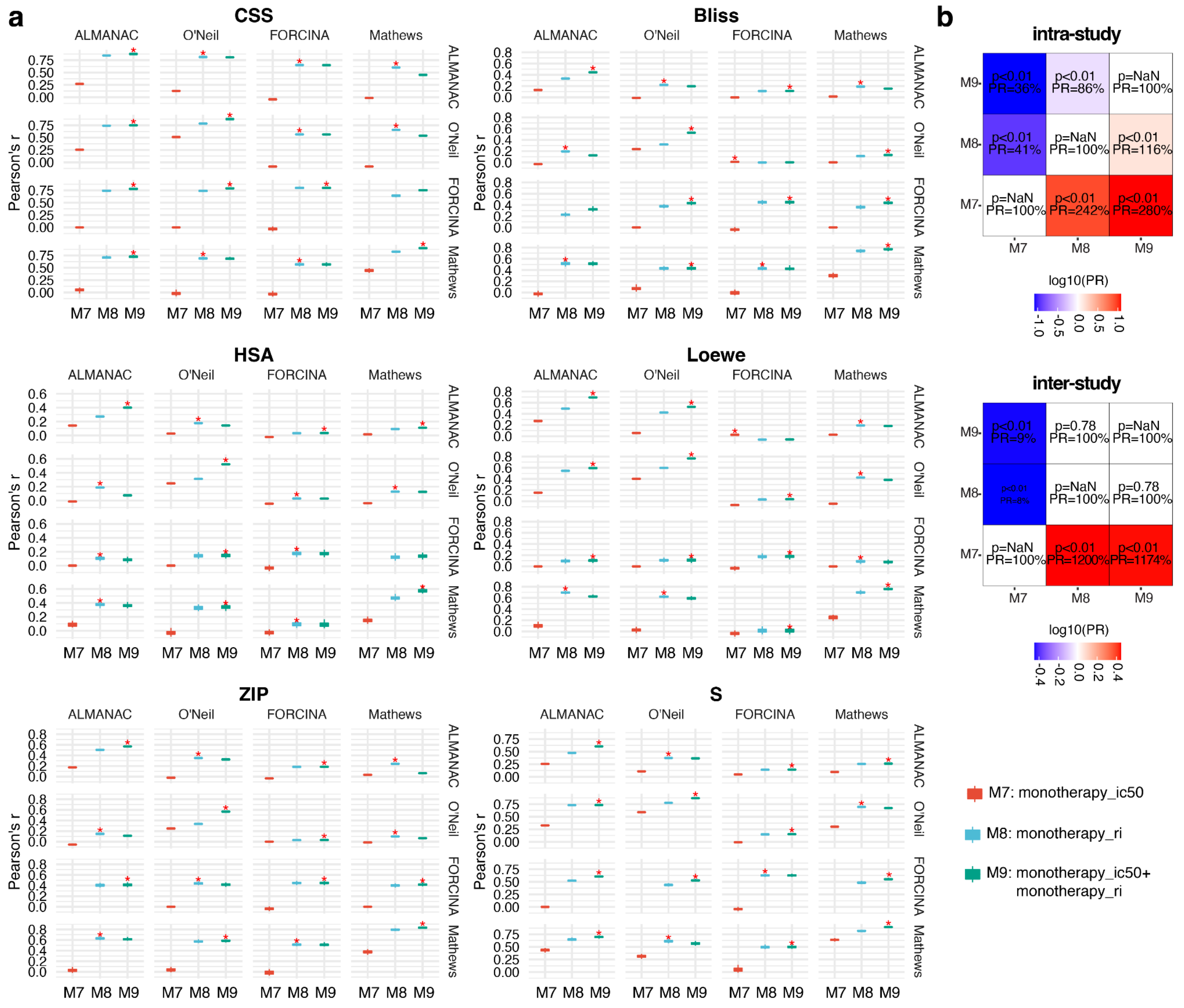

Supplementary Figure 8. Pair-wise comparison of model performances over each combination treatment response score (CSS, Bliss, HSA, Loewe, ZIP, S) using p-values from paired t-test and performance ratios (PR). The models in this figure are corresponding to Supplementary Figure 7 B. A. intra-study cross-validation. B. inter-study cross-validation.

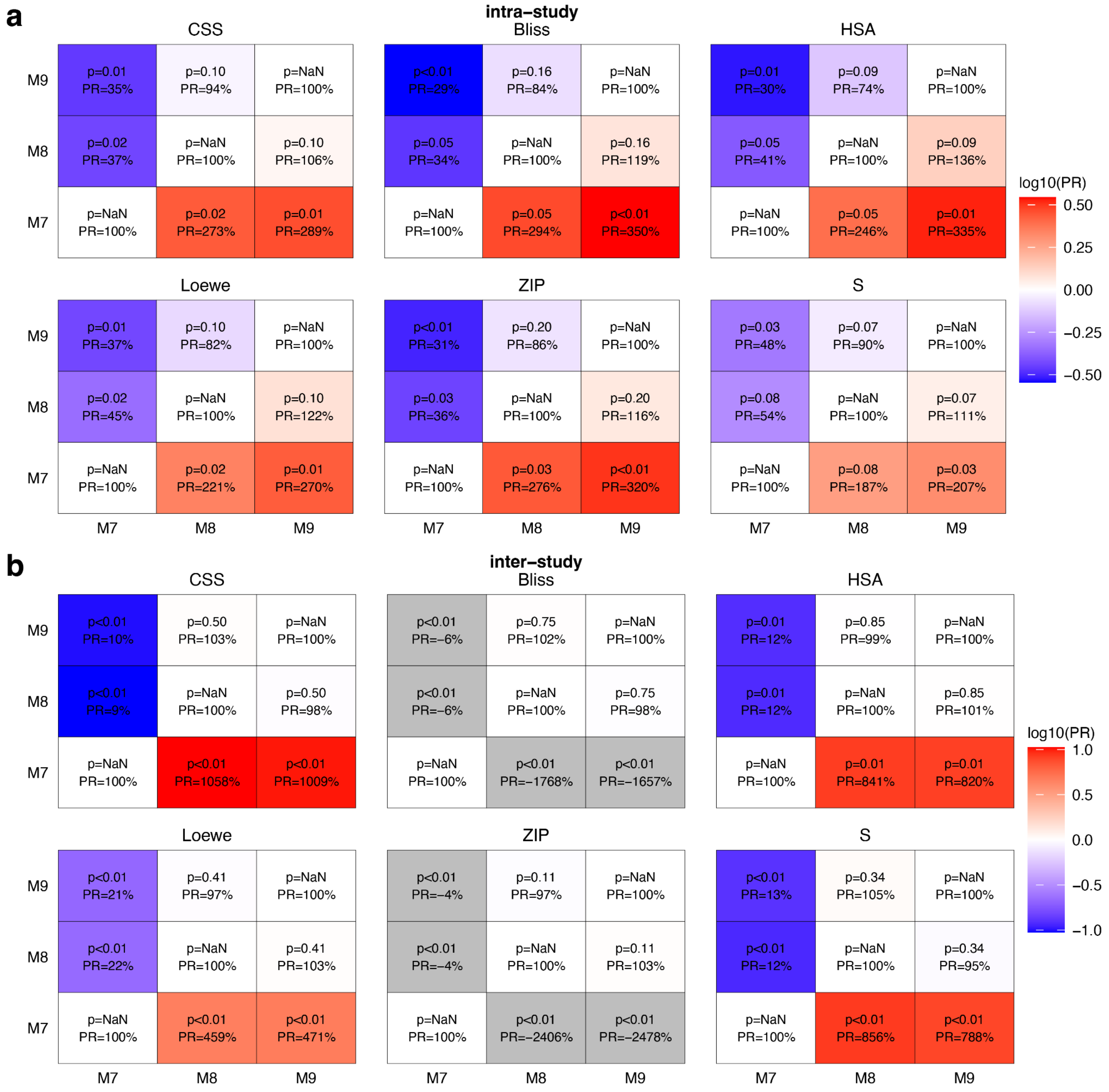

Supplementary Figure 9. Comparison between monotherapy efficacy model and in monotherapy efficacy model in addition to different drc models. A. Performances of all interpolation models in different training (top) and testing (right) settings. C. Comparison of performances between models by paired t-test and performance ratio (PR) on the average.

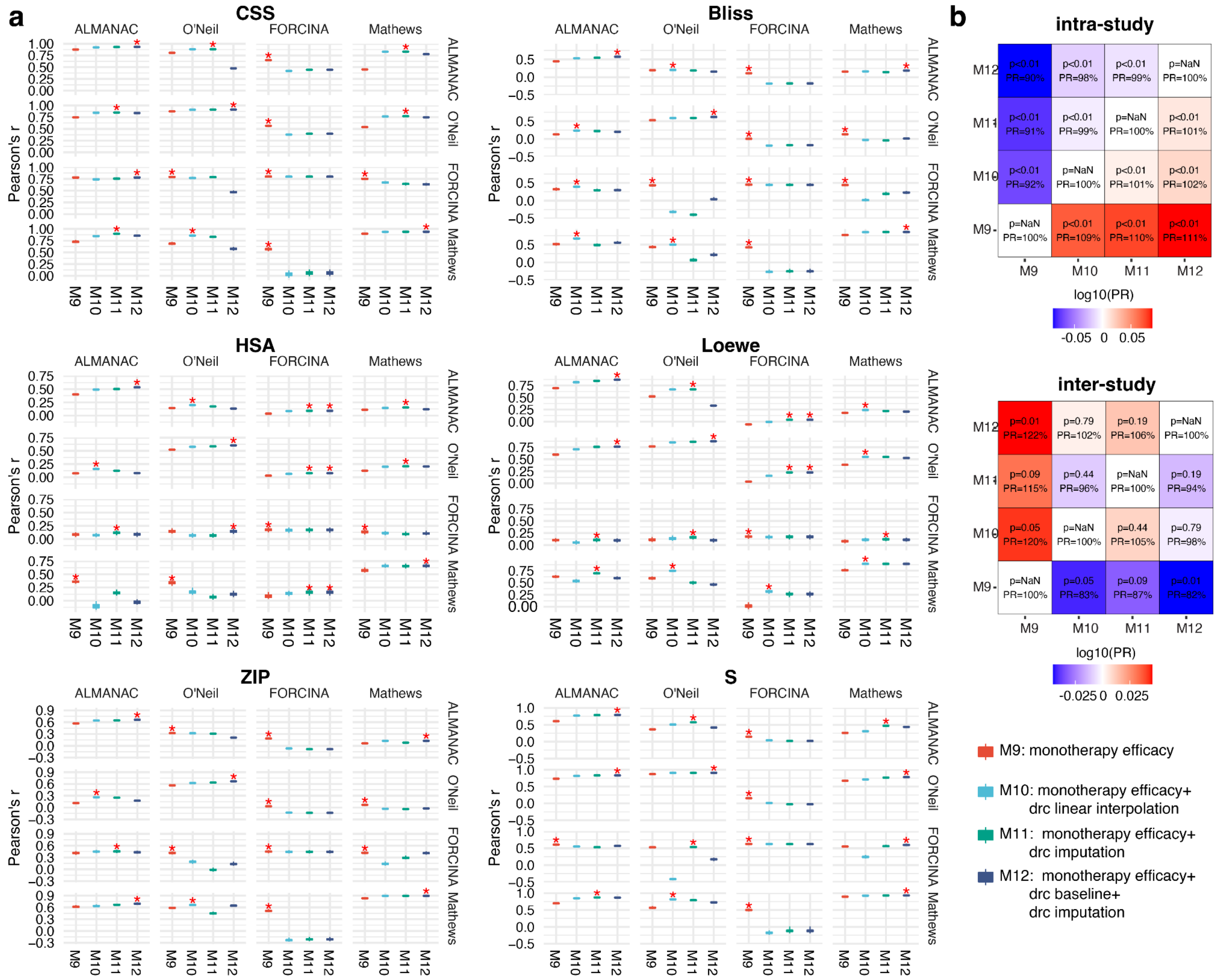

Supplementary Figure 10. Pair-wise comparison of model performances over each combination treatment response score (CSS, Bliss, HSA, Loewe, ZIP, S) using p-values from paired t-test and performance ratios (PR). The models in this figure are corresponding to Supplementary Figure 9 B. A. intra-study cross-validation. B. inter-study cross-validation.

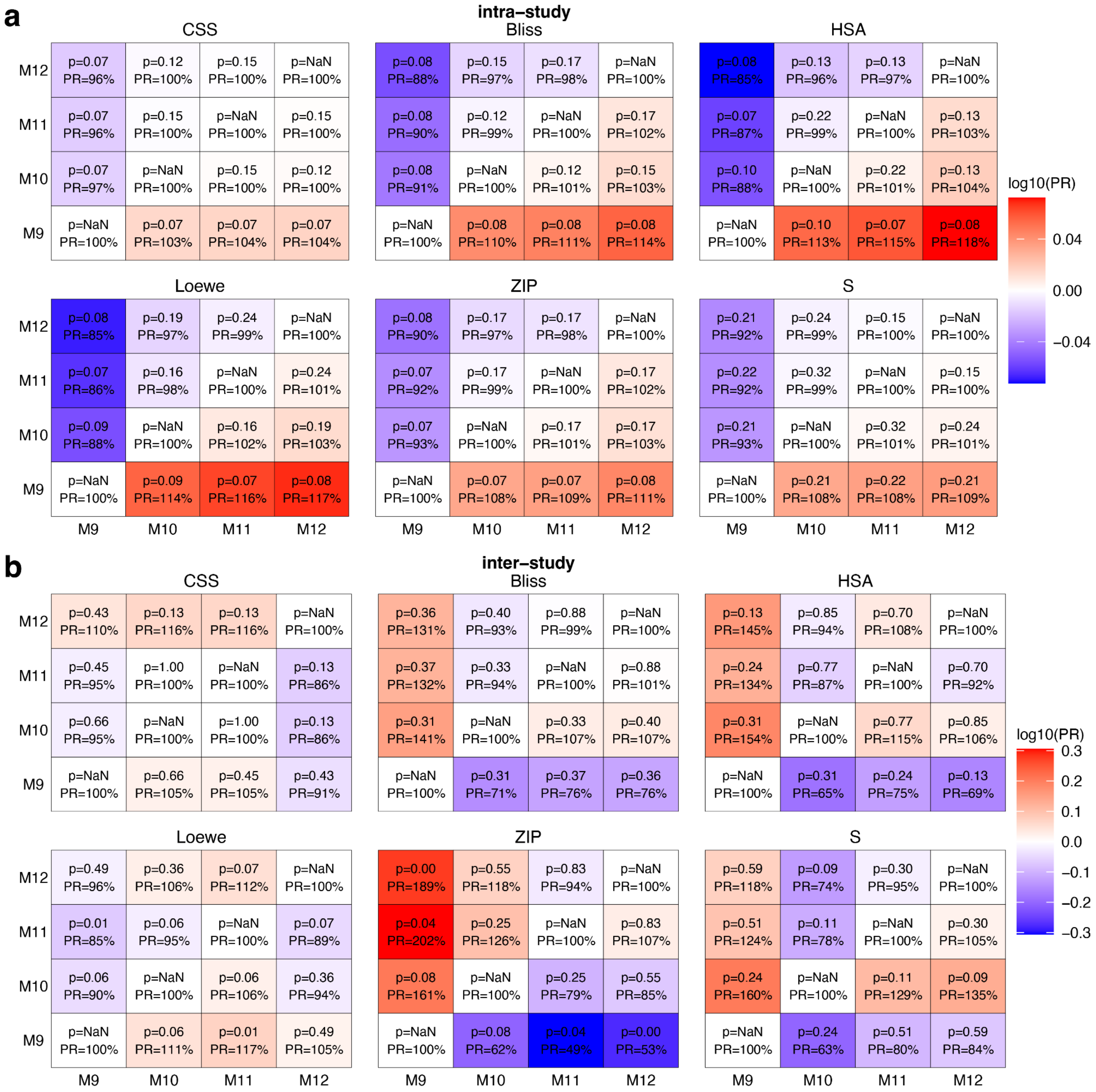

Supplementary Figure 11. Pair-wise comparison of model performances over each combination treatment response score (CSS, Bliss, HSA, Loewe, ZIP, S) using p-values from paired t-test and performance ratios (PR). The models in this figure are corresponding to Figure 3C. A. intra-study cross-validation. B. inter-study cross-validation.

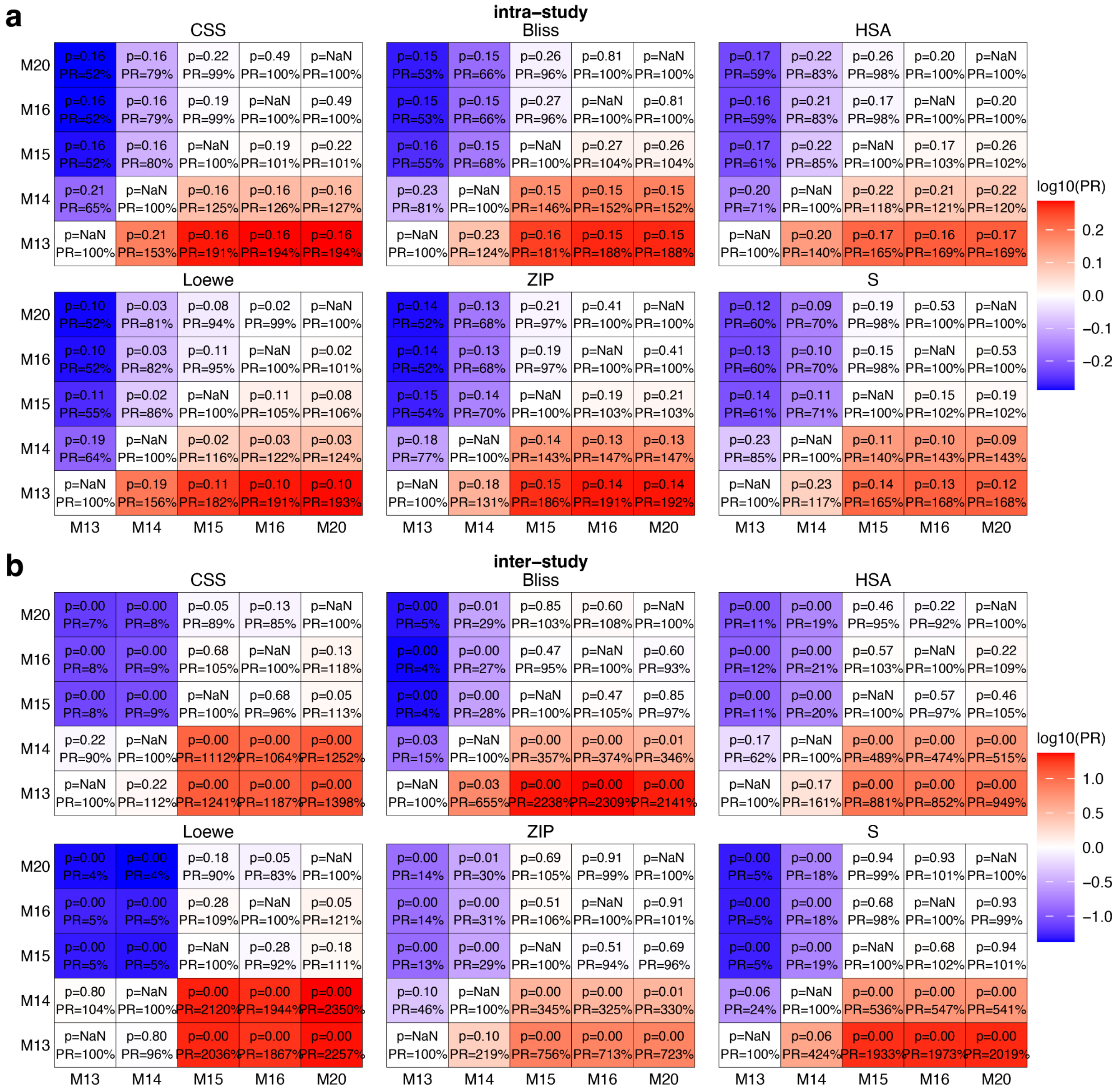

Supplementary Figure 12. Feature contribution of best performing model (M20 in Figure 3) in inter-study prediction when trained on ALMANAC and tested on O'Neil study. The importance when predicting all six response scores were shown below.

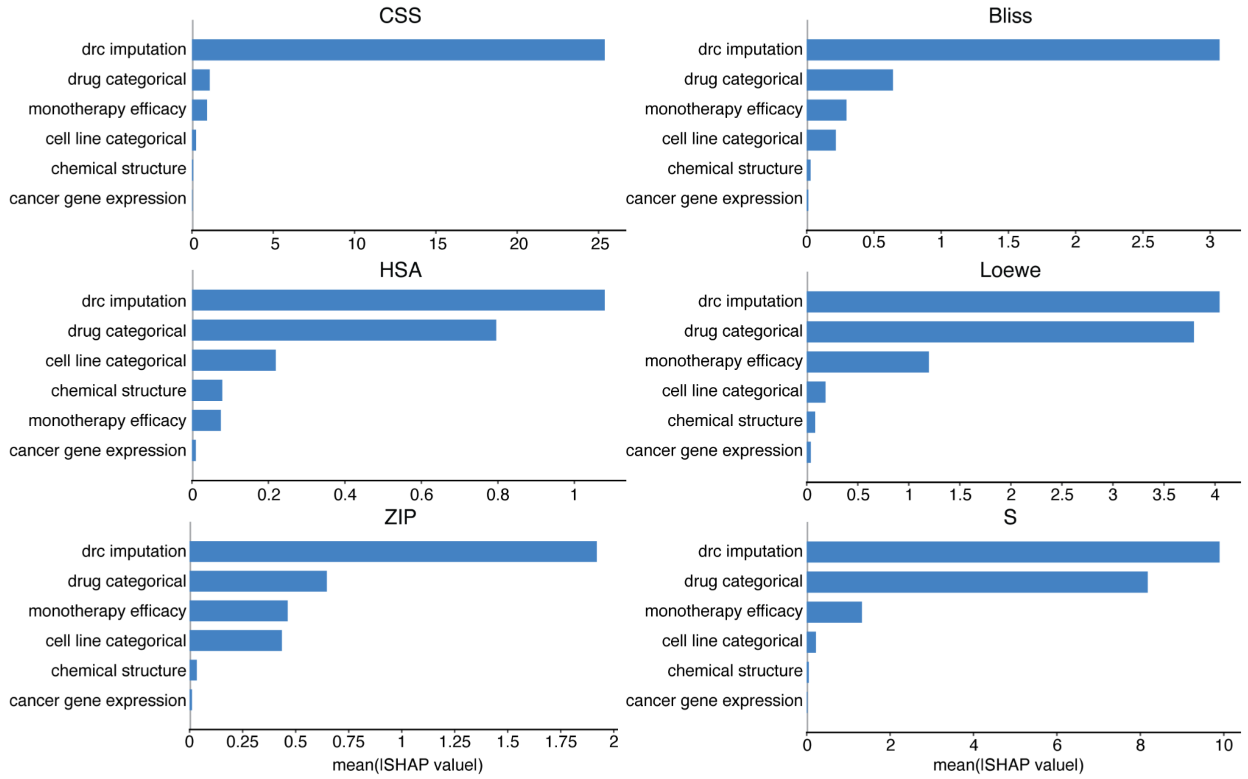

Supplementary Figure 13. Feature contribution of dose-response curve imputation features (M12) in inter-study CSS score prediction when trained on ALMANAC and tested on O'Neil study. A. summary plot of ten imputation features. B. Bar plot shows the contribution (average impact in model output magnitude) of ten imputation features. C. scatter plot shows the relationship between feature value and contribution (SHAP value) of 10 imputation features.

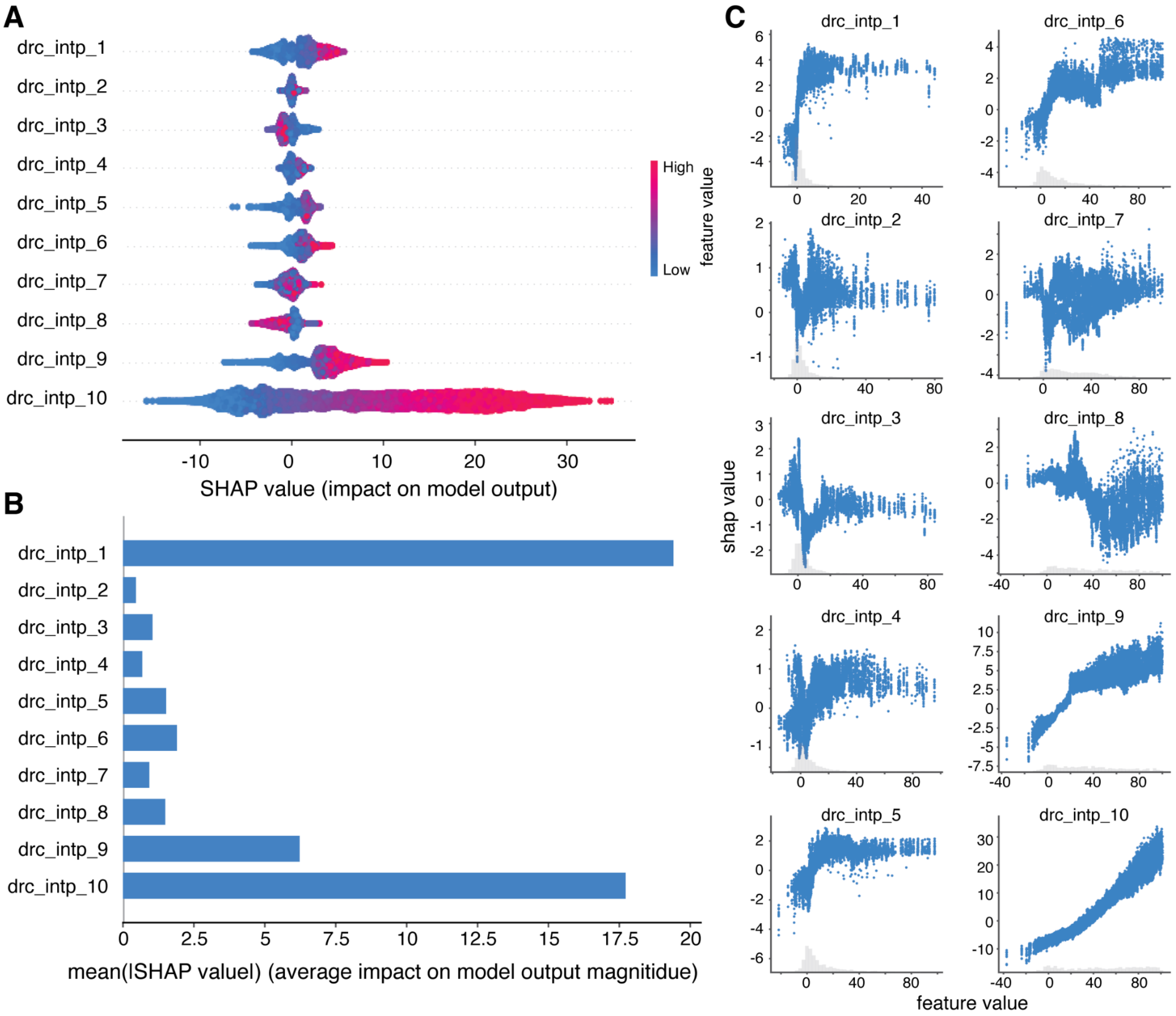

Supplementary Figure 14. Comparison between different dose-response curve interpolation methods in 3 vs. 1 inter-study cross-validation. A. Performances of all interpolation models in different training (top) and testing (right) settings. C. Comparison of performances between models by paired t-test and performance ratio (PR) on the average. The models in this figure are the same as **Supplementary Figure 5.**

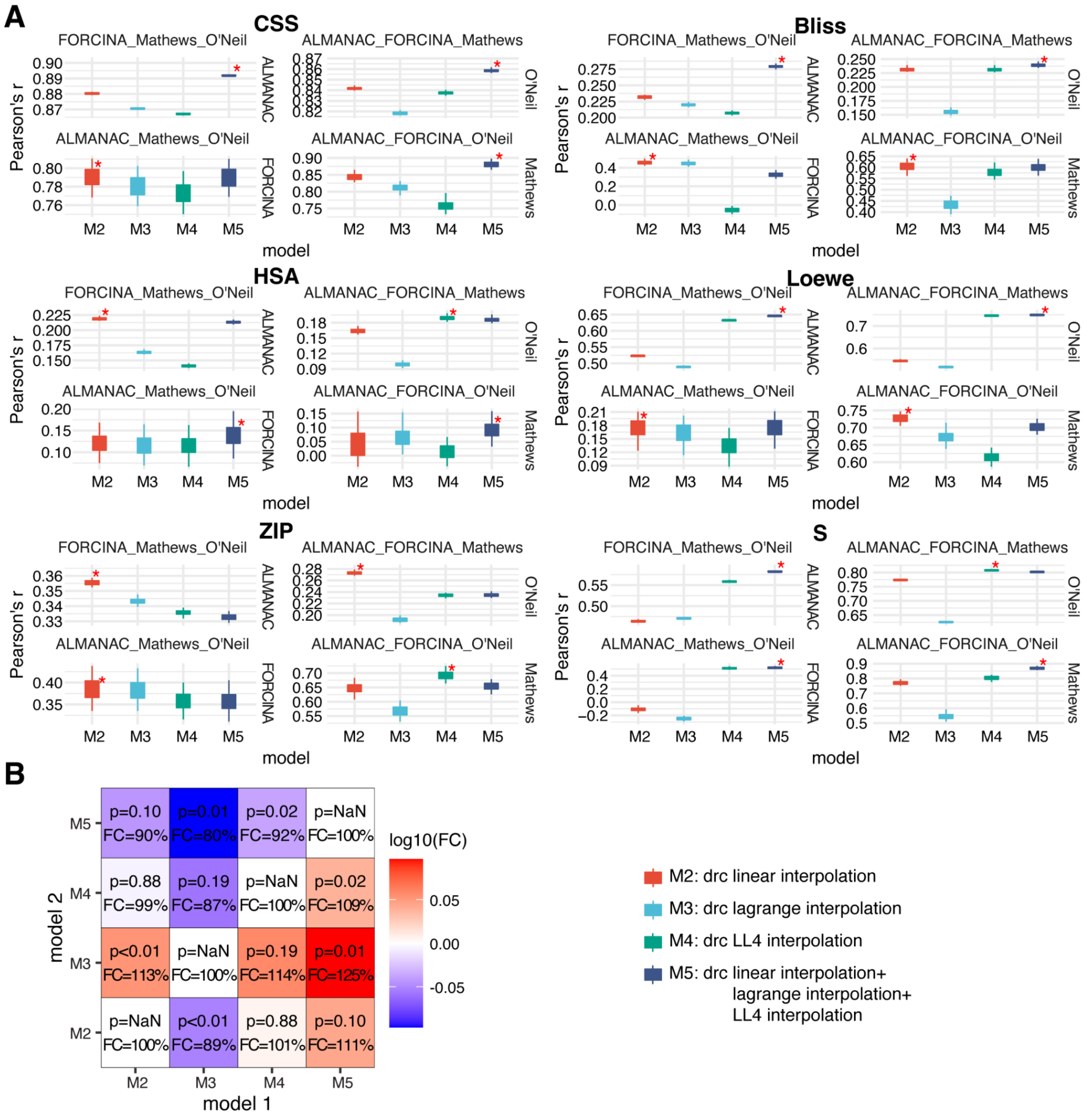

Supplementary Figure 15. Comparison between different monotherapy efficacy features in 3 vs. 1 inter-study cross-validation. A. Performances of all monotherapy efficacy models in different training (top) and testing (right) settings. C. Comparison of performances between models by paired t-test and performance ratio (PR) on the average. The models in this figure are the same as **Supplementary Figure 7.**

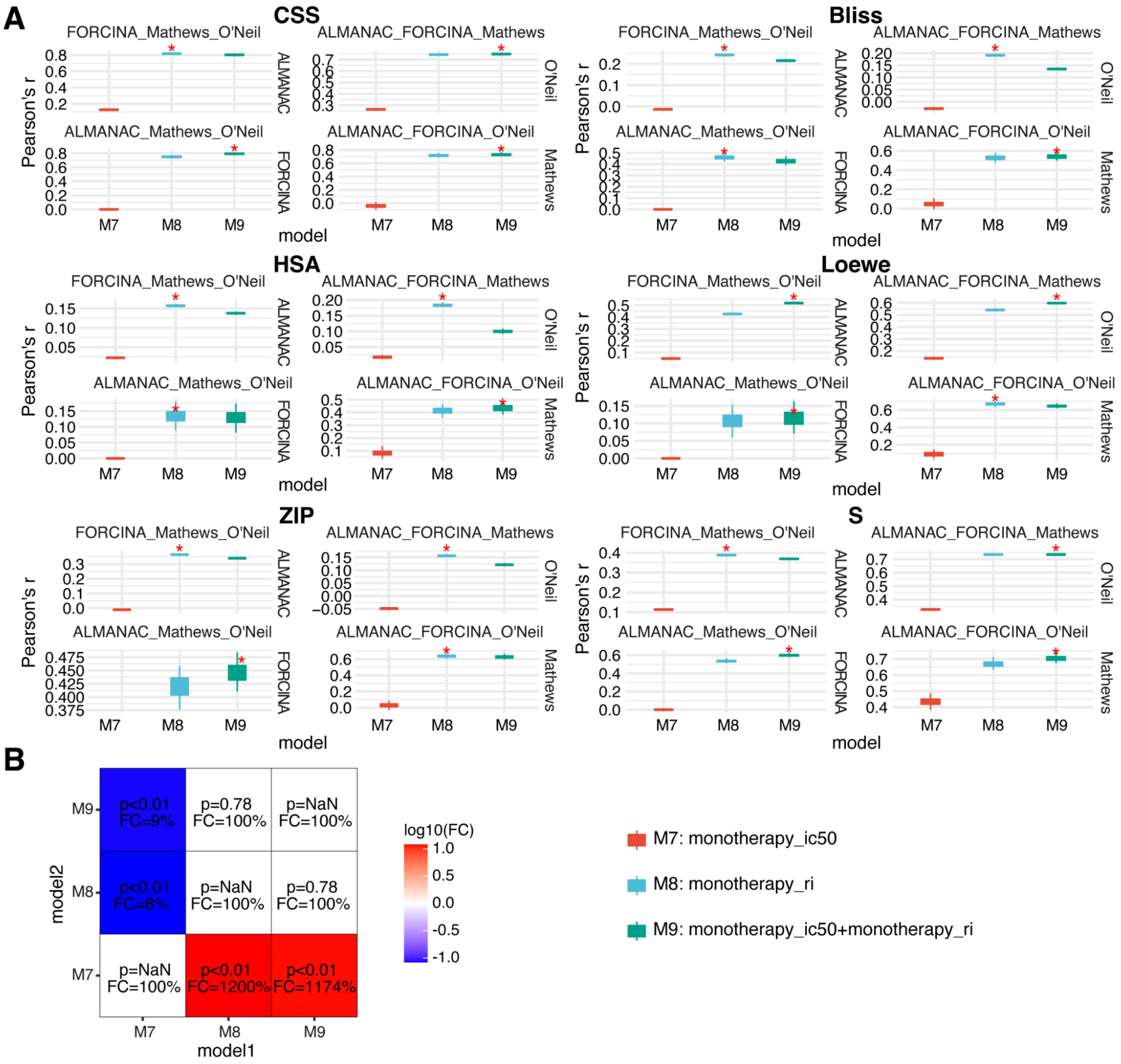

Supplementary Figure 16. Comparison between monotherapy efficacy features in addition to different dose-response curve features in 3 vs. 1 inter-study cross-validation. A. Performances of all models in different training (top) and testing (right) settings. C. Comparison of performances between models by paired t-test and performance ratio (PR) on the average. The models in this figure are the same as **Supplementary Figure 9.**

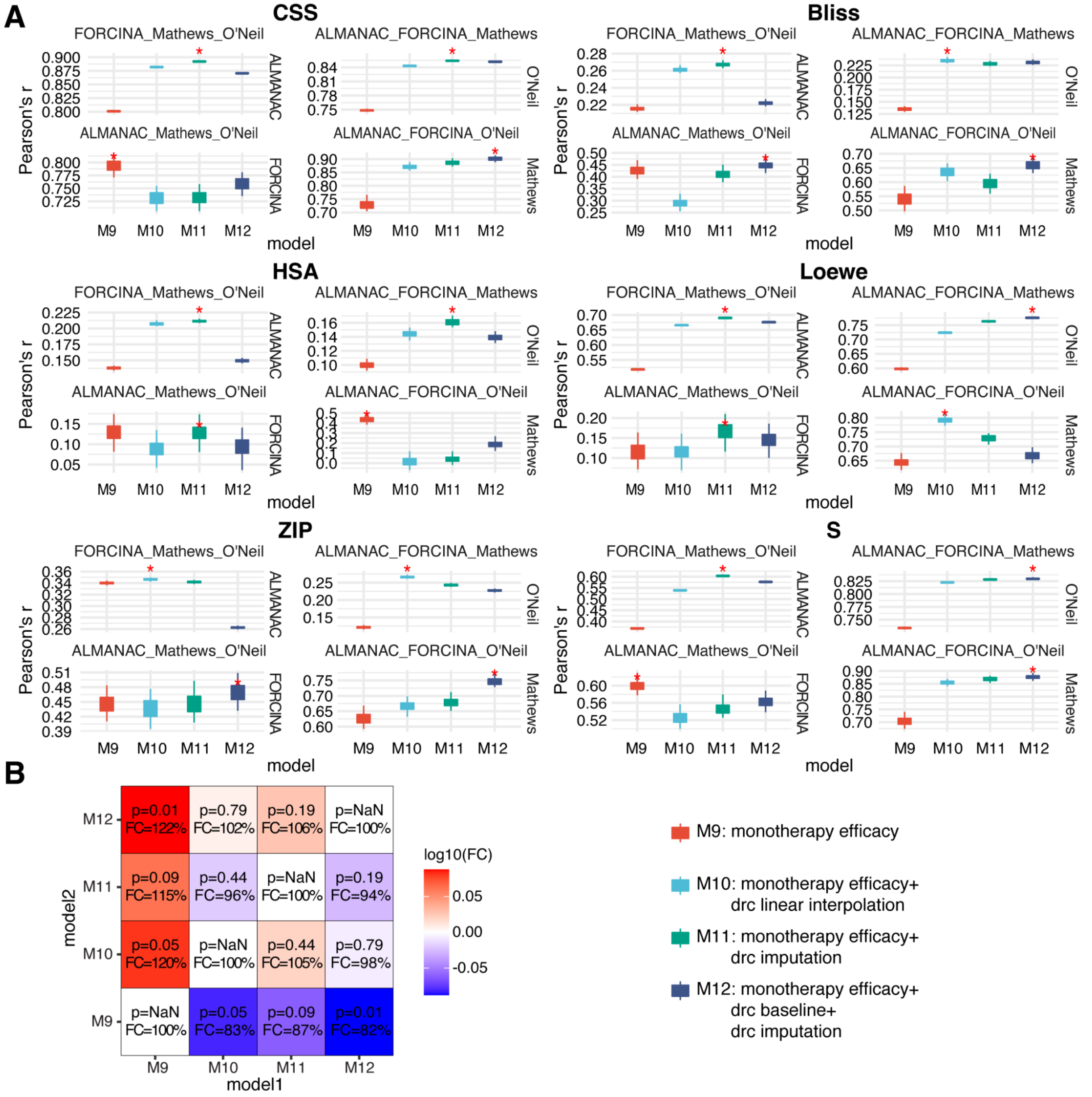

Supplementary Figure 17. Comparison between different combinations of pharmacological features in 3 vs. 1 inter-study cross-validation. A. Performances of all monotherapy efficacy models in different training (top) and testing (right) settings. C. Comparison of performances between models by paired t-test and performance ratio (PR) on the average. The models in this figure are the same as **Figure 2.**

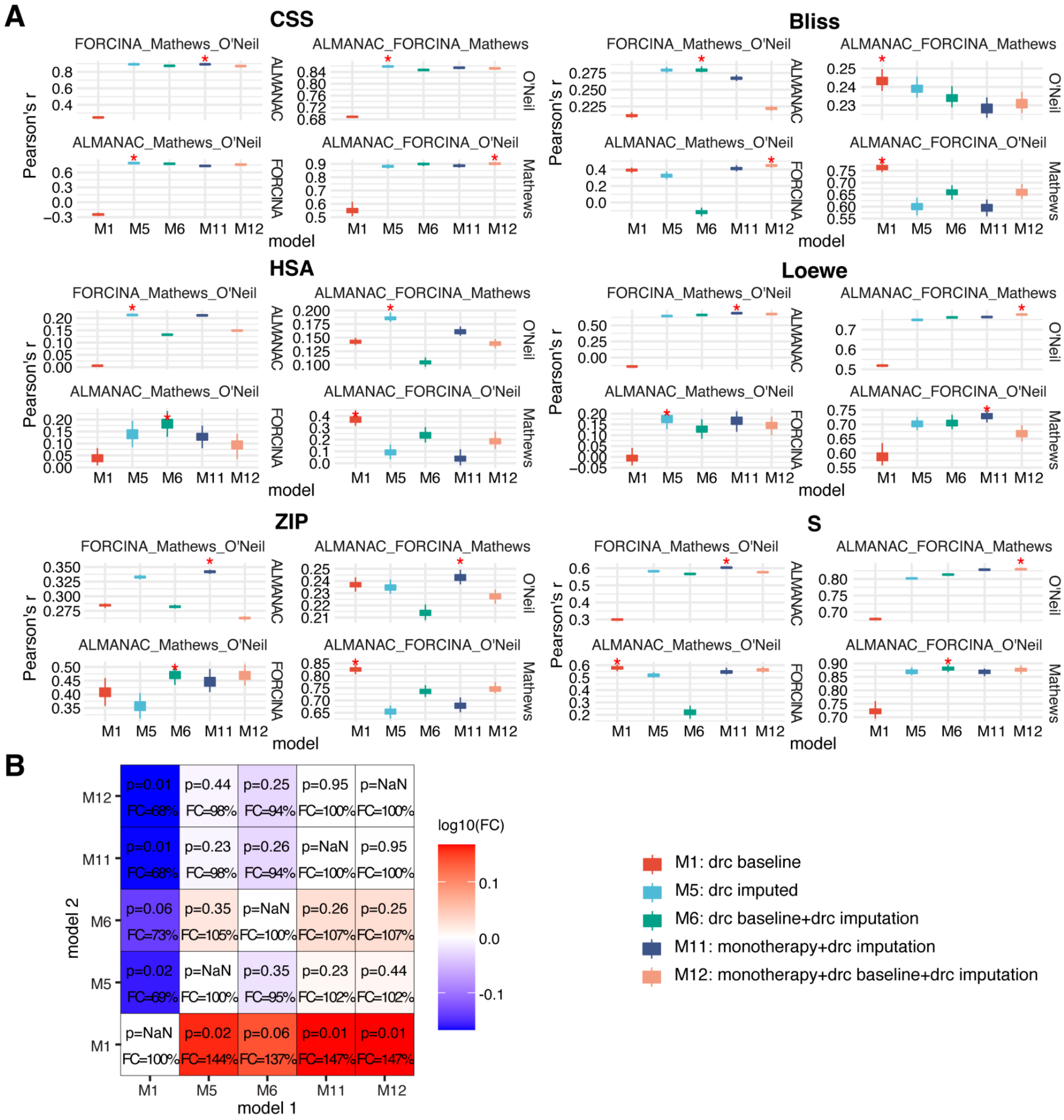

Supplement: Supplementary file 2 — Supplementary Information [file 42003_2023_4783_MOESM2_ESM.pdf]
