# Supplementary material for: Harmonizing across datasets to improve the transferability of drug combination prediction
Source: Commun Biol. 2023 Apr 11;6:397. doi: 10.1038/s42003-023-04783-5 (PMC10090076; doi:10.1038/s42003-023-04783-5)
Supplement: Supplementary file 3 — Reporting Summary [file 42003_2023_4783_MOESM3_ESM.pdf]

nature portfolio

Corresponding author(s): *Double-blind peer review submissions: write DBPR and your manuscript number here instead of author names.*

Last updated by author(s): *YYYY-MM-DD*

Reporting Summary

Nature Portfolio wishes to improve the reproducibility of the work that we publish. This form provides structure for consistency and transparency in reporting. For further information on Nature Portfolio policies, see our Editorial Policies and the Editorial Policy Checklist.

Statistics

For all statistical analyses, confirm that the following items are present in the figure legend, table legend, main text, or Methods section.

n/a	Confirmed	
☐	☒	The exact sample size (*n*) for each experimental group/condition, given as a discrete number and unit of measurement
☐	☒	A statement on whether measurements were taken from distinct samples or whether the same sample was measured repeatedly
☐	☒	The statistical test(s) used AND whether they are one- or two-sided<br>*Only common tests should be described solely by name; describe more complex techniques in the Methods section.*
☐	☒	A description of all covariates tested
☐	☒	A description of any assumptions or corrections, such as tests of normality and adjustment for multiple comparisons
☐	☒	A full description of the statistical parameters including central tendency (e.g. means) or other basic estimates (e.g. regression coefficient) AND variation (e.g. standard deviation) or associated estimates of uncertainty (e.g. confidence intervals)
☐	☒	For null hypothesis testing, the test statistic (e.g. *F*, *t*, *r*) with confidence intervals, effect sizes, degrees of freedom and *P* value noted<br>*Give P values as exact values whenever suitable.*
☐	☒	For Bayesian analysis, information on the choice of priors and Markov chain Monte Carlo settings
☐	☒	For hierarchical and complex designs, identification of the appropriate level for tests and full reporting of outcomes
☐	☒	Estimates of effect sizes (e.g. Cohen's *d*, Pearson's *r*), indicating how they were calculated

Our web collection on statistics for biologists contains articles on many of the points above.

Software and code

Policy information about availability of computer code

Data collection	Currently, DrugComb has been the largest public data portal for in vitro high-throughput combination treatment screening studies. We selected the four largest datasets (ALMANAC, O'Neil, FORCINA, and Mathews) from DrugComb (https://drugcomb.org/) for the inter and cross-study analysis in this paper, where the detailed comparisons for the four datasets are shown in Supplementary Table 1.<br>DrugComb provides six metrics (CSS, S, Bliss, HSA, ZIP, Loewe) for the responses of combination treatments, and two metrics (IC50 and RI (relative inhibition)) for the response of single drug treatments. The details of the formula of these metrics have been described in Zheng et al. 8. Briefly, CSS analyzes the overall drug efficacy for the combination treatment, while S, Bliss, HSA, ZIP, and Loewe evaluate the synergy or the degree of interaction between the two drugs used in a combination treatment. Besides the efficacy and synergy metrics for monotherapy/combination therapy, DrugComb also provides the SMILES (Simplified molecular input line entry system) format chemical structure of drugs, which is used for structural encoding in this study.<br>The transcriptomic profiles of all the cancer cell lines used in this study were obtained from CCLE (Cancer Cell Line Encyclopedia) (https://sites.broadinstitute.org/ccle/datasets). We obtained 279 cancer-associated genes from the IMPACT (Integrated Mutation Profiling of Actionable Cancer Targets) project 33, 273 of which were found to be overlapped with the CCLE transcriptomic profiles. Therefore, these 273 genes were used for combination treatment response prediction in this study.
Data analysis	The source code of the analysis and models are available on GitHub: https://github.com/GuanLab/DrugComb-cross-study-prediction.

For manuscripts utilizing custom algorithms or software that are central to the research but not yet described in published literature, software must be made available to editors and reviewers. We strongly encourage code deposition in a community repository (e.g. GitHub). See the Nature Portfolio guidelines for submitting code & software for further information.

Data

Policy information about availability of data

All manuscripts must include a data availability statement. This statement should provide the following information, where applicable:

- Accession codes, unique identifiers, or web links for publicly available datasets
- A description of any restrictions on data availability
- For clinical datasets or third party data, please ensure that the statement adheres to our policy

The data analyzed in this study can be freely downloaded from DrugComb data portal: https://drugcomb.fimm.fi/.

Human research participants

Policy information about studies involving human research participants and Sex and Gender in Research.

Reporting on sex and gender	No human participants has involved in our study, therefore sex and gender information is not collected.
Population characteristics	See above.
Recruitment	No human participants were recruited.
Ethics oversight	No approval was needed since no human participants is involved.

Note that full information on the approval of the study protocol must also be provided in the manuscript.

Field-specific reporting

Please select the one below that is the best fit for your research. If you are not sure, read the appropriate sections before making your selection.

☒ Life sciences ☐ Behavioural & social sciences ☐ Ecological, evolutionary & environmental sciences

For a reference copy of the document with all sections, see nature.com/documents/nr-reporting-summary-flat.pdf

Life sciences study design

All studies must disclose on these points even when the disclosure is negative.

Sample size	We explore the current latest version of the DrugComb portal (https://drugcomb.org/), which contains the most comprehensive publicly-available drug combination high-throughput screening datasets, including 24 independent studies. Among them, we select four major datasets: ALMANAC, O'Neil, FORCINA, and Mathews, as they are of the biggest sizes and therefore are commonly used in machine learning prediction of combination responses These four studies contain a total of 406,479 drug combination experiments, 9,163 drugs, and 92 cell lines.
Data exclusions	Data with missing cell line/drug/drug response information was excluded from our analysis.
Replication	O'Neil has the best quality, where all the combinations are tested with four replicates, whereas ALMANAC tested at most three replicates for each combination and Mathews tested two replicates for each combination. In contrast, the FORCINA dataset contains no replicates.
Randomization	We conducted cross-validation within each of the dataset with random splits on drug-cell line combinations. The cross-validation was set up so that the training and testing sets in this step do not share the same treatment-cell line combinations.
Blinding	Blinding was not relevant to our study as datasets during cross-validation are randomly split.

Reporting for specific materials, systems and methods

We require information from authors about some types of materials, experimental systems and methods used in many studies. Here, indicate whether each material, system or method listed is relevant to your study. If you are not sure if a list item applies to your research, read the appropriate section before selecting a response.

Materials & experimental systems

n/a	Involved in the study	
☒	☐	Antibodies
☒	☐	Eukaryotic cell lines
☒	☐	Palaeontology and archaeology
☒	☐	Animals and other organisms
☒	☐	Clinical data
☒	☐	Dual use research of concern

Methods

n/a	Involved in the study	
☒	☐	ChIP-seq
☒	☐	Flow cytometry
☒	☐	MRI-based neuroimaging
